# Novel endogenous protein-based strategies to inhibit clinically relevant bacterial AB-type toxins including pertussis toxin

**DOI:** 10.1007/s00204-026-04372-5

**Published:** 2026-04-17

**Authors:** Stefanie Lietz, Holger Barth

**Affiliations:** Institute of Experimental and Clinical Pharmacology, Toxicology and Pharmacology of Natural Products, Ulm University Medical Center, 89081 Ulm, Germany

**Keywords:** *Bordetella pertussis*, Pertussis toxin, AB-type protein toxin, Toxin inhibitor, Endogenous proteins, Endogenous peptides, Antimicrobial peptides, α_1_-antitrypsin, Human defensins, Human serum albumin

## Abstract

Many highly infectious and severe diseases such as pertussis, diphtheria, anthrax, and *Clostridioides difficile* infections are caused by bacteria which release symptom-inducing AB-type protein toxins. These diseases are treated using antibiotics or preventive measures including vaccination. However, despite preventive measures, increasing case numbers have been reported in past years. Therefore, novel therapeutic options against toxin-mediated disease are urgently required. Since the toxins are the causative agents of the diseases, the development of pharmacological inhibitors that specifically neutralize individual toxins should be a relevant strategy to further support the current therapies. Huge potential to identify toxin inhibitors lies within the human proteome/peptidome that might contain endogenous toxin inhibitors as yet unknown part of the innate immunity. Screening of human peptide libraries and systematic testing of proteins/peptides with antimicrobial activity led to the identification of proteins/peptides with specific anti-toxin activities. Consequently, defensins, α_1_-antitrypsin and derived peptides, human serum albumin, and in silico predicted angiogenin-derived peptides were identified as potent inhibitors for bacterial toxins including *Clostridioides difficile* toxins TcdA, TcdB and CDT, diphtheria, anthrax, and pertussis toxin, and clostridial binary iota and C2 toxins. This review summarizes the current state of identified endogenous proteins/peptides as novel inhibitors for clinically relevant bacterial AB-type toxins.

## Introduction

Many life-threatening infectious diseases caused by pathogenic bacteria are directly linked to the presence of AB-type protein toxins that are secreted as main virulence factors by the bacteria. AB-type toxins efficiently enter mammalian target cells and act intracellularly as enzymes. Therefore, AB-type toxins contain functionally different subunits, namely an enzymatically active A-subunit and a binding/translocation B-subunit. The latter mediates receptor-binding and endocytic uptake of the toxin into cellular compartments. Subsequently, the B-subunit facilitates the translocation of the A-subunit from the cellular compartment into the cytosol where the A-subunit modifies its corresponding substrate with high specificity and potency. This disrupts cellular structures and/or associated signal-transduction cascades which ultimately contributes to the development of clinical symptoms characteristic for the respective disease.

**Fig. 1:**
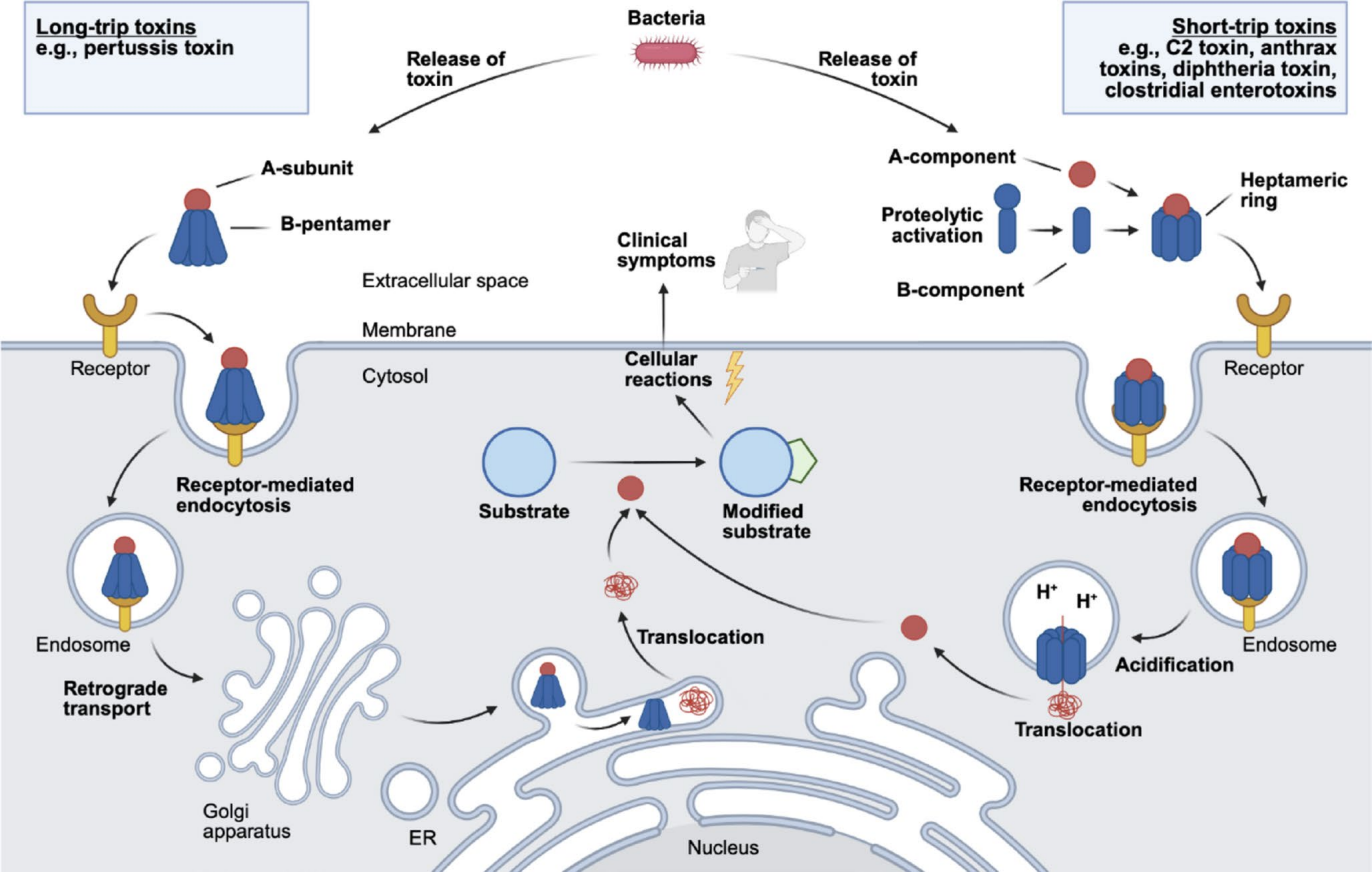
Different uptake routes of short- and long-trip AB-type protein toxins into mammalian target cells. Long-trip toxins (left) are taken up via receptor-mediated endocytosis and follow a retrograde transport route from the Golgi apparatus to the endoplasmic reticulum (ER). Within the ER, the toxin gets dissembled, while the enzyme domain is translocated into the cytosol where the modification of its specific substrate takes place. In contrast to long-trip toxins, short-trip toxins (right, exemplarily shown for the binary short-trip toxins, e.g., C2 toxin) translocate the enzyme component into the cytosol of target cells after the acidification of endosomes. For both, long- and short-trip toxins, the uptake which triggers the modification of the corresponding substrate, cellular reactions occur that are associated with the symptoms of the respective toxin-mediated disease. (Created in BioRender. Lietz, S. (2026) https://BioRender.com/d0i8prn)

Bacterial AB-type toxins can be further divided into two larger groups regarding to their distinct intracellular uptake routes, namely the short- and long-trip toxins (Fig. 1). Short-trip toxins deliver their A-subunit from acidified endosomal vesicles into the cytosol, while long-trip toxins follow a retrograde route from endosomes via the Golgi apparatus to the endoplasmic reticulum (ER), from where their A-subunits are translocated into the cytosol.

For some toxin-mediated diseases, preventive measures, such as vaccinations are available. However, despite vaccinations, recent outbreaks of e.g., pertussis and diphtheria have been reported, aligning with increasing case numbers, also in western countries (European Centre for Disease Prevention and Control, 2023; European Centre for Disease Prevention and Control., 2024). Therefore, toxin-associated diseases are still a matter of concern, not least due to the limitation of therapeutic options. Those are often restricted to the treatment of symptoms and the use of antibiotics, which in case of e.g., pertussis requires early onset application for a positive outcome of the patients’ disease progression (Mattoo and Cherry 2005). This is explained by the fact that antibiotics only efficiently eliminate the toxin-producing bacteria, but whatsoever are ineffective against secreted AB-type toxins. Moreover, increasing resistance of bacterial pathogens against antibiotics and immune evasion challenge the treatment of toxin-associated diseases in the clinics and the development of efficient vaccines (Ma et al. 2021). Therefore, novel therapeutic options that directly target the disease provoking factors, i.e. the toxins, are urgently required. A huge potential for the discovery of novel toxin-neutralizing molecules lies within the human proteome and peptidome (Bosso et al. 2018). We and others discovered various body-own proteins and peptides that neutralize clinically relevant bacterial AB-type toxins including pertussis toxin, diphtheria toxin, anthrax toxin, and clostridial enterotoxins. This is reviewed here, with a special focus on pertussis toxin.

## Pertussis toxin and whooping cough–uptake, mode of action, and disease pathology

**Fig. 2:**
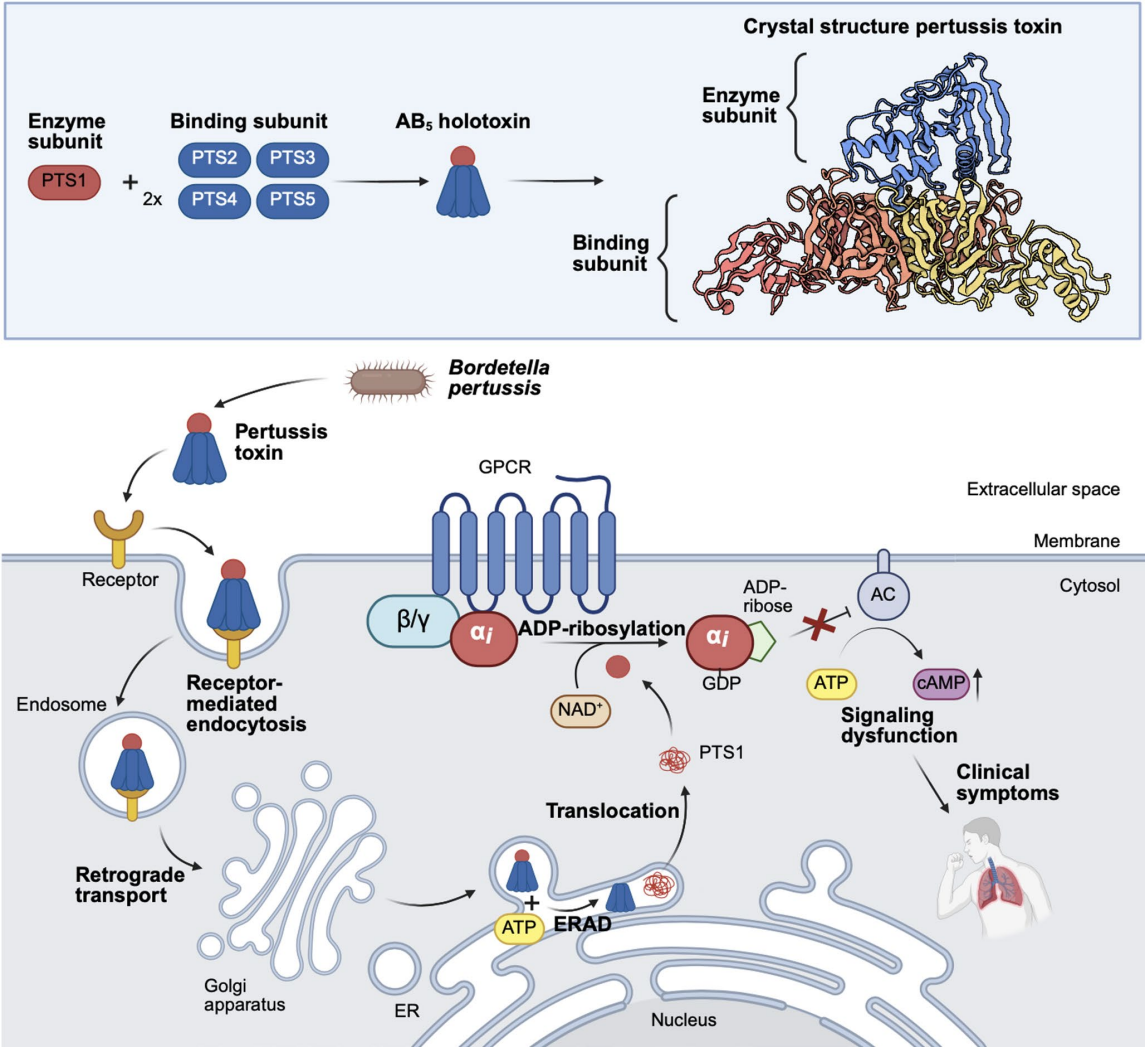
Structure and uptake of the AB_5_ holotoxin PT from *B. pertussis*. Pertussis toxin (PT) which is released from *B. pertussis* is an AB_5_ holotoxin and consists of one enzyme subunit S1 (PTS1) and five binding subunits S2, S3, S4, and S5 (PTS2-5) which assemble in a 1:1:1:2:1 stoichiometry (upper part, PDB code: 1PRT, (Stein et al. 1994). After receptor binding of PT, receptor mediated endocytosis occurs. Next, PT toxin follows a retrograde transport from the endosomes via the Golgi apparatus to the endoplasmic reticulum (ER). Within the ER, PT gets disassembled, while the enzyme subunit PTS1 is transported by the ER associated degradation pathway (ERAD) into the cytosol. Here, PTS1 modifies and thus ADP-ribosylates its substrate, inhibitory G-proteins (Gαi) of G-protein-coupled receptors (GPCR), while NAD^+^ serves as a co-substrate. As such Gαi is no longer able to downregulate the adenylate cyclase (AC) activity which contributes to the accumulation of intracellular cAMP. (adenosine triphosphate (ATP), adenosine diphosphate (ADP), cyclic adenosine monophosphate (AMP) (cAMP), guanosine diphosphate (GDP) (According to (Hoonakker 2021; Lietz et al. 2024a, b; Nowakowska-Gołacka et al. 2019; Stein et al. 1994; Teter 2019), (Created in BioRender. Lietz, S. (2025) https://BioRender.com/k01ltmv)

Pertussis toxin (PT) is one of the main secreted virulence factors of the bacterium *Bordetella (B.) pertussis* and contributes to the disease pathology of the corresponding highly infectious respiratory disease pertussis *alias* whooping cough. Despite that pertussis belongs to the vaccine-preventable infectious diseases of the human lung, increasing case numbers have been reported recently by the European Centre for Disease Prevention and Control (ECDC). The annual pre-COVID-19-pandemic case numbers of pertussis with e.g., 41 026 cases in 2016 and 34 468 cases in 2019, were almost exceeded with over 32 000 reported cases in the EU/EEA countries already in the first three months of 2024 (European Centre for Disease Prevention and Control., 2024). The extensive vaccination programs failed to achieve full vaccination coverage, resulting in unprecedented levels of pertussis with a novel high in reported cases, especially in the age groups of < 1 to 19 years, while the strongest increase was observed for 10–14 year olds in EU/EEA countries (European Centre for Disease Prevention and Control., 2024; Locht and Antoine 2021; Yeung et al. 2017). Certainly, increased testing for pertussis, waning immunity, and at EU/EEA level a deceased vaccination coverage from 97% in 2012 to 94% in 2022, contributed to the reported increase of pertussis cases (European Centre for Disease Prevention and Control., 2019, 2024; Kaczmarek et al. 2013). Predominantly, vulnerable groups suffer from severe cases of pertussis which includes infants younger than three months who are too young to be vaccinated (Mattoo and Cherry 2005). The severe pertussis disease can ultimately cause death, but is also accompanied by other characteristic complications, including the typical long-lasting paroxysmal cough or leukocytosis, pneumonia, encephalopathy, seizures, and apnea which may require hospitalization of the patient (Mattoo and Cherry 2005; Surridge et al. 2007). In addition, severe pertussis is associated with the important virulence factor of *B. pertussis*, PT, which belongs to the AB-type protein toxins with ADP-ribosyltransferase (ART) activity (Mattoo and Cherry 2005; Scanlon et al. 2019). The pertussis AB_5_ holotoxin consists of an enzymatically active A-subunit (PTS1) and a pentameric ring structure B-subunit (PTS2-5), while PT possesses a 1:1:1:2:1 stoichiometry of the S1:S2:S3:S4:S5 subunits (Fig. 2) (Stein et al. 1994; Tamura et al. 1982; Weiss et al. 1993). Via the binding B-subunit, PT binds to sialic acid conjugates on target cells which enables the uptake of PT into endosomes via receptor-mediated endocytosis (Armstrong et al. 1988; Witvliet et al. 1989). From the endosomes, PT travels retrogradely via the Golgi apparatus to the ER (el Bayâ et al. 1997; Plaut and Carbonetti 2008). Here, PT disassembles and releases the PTS1 subunit, due to the attachment of ATP to the central pore of the B-pentamer which originates from the ATP stores of the ER. Since the from the B-subunit separated PTS1 is thermally unstable, it shifts into a disordered/unfolded state which is recognized by the ER associated degradation pathway (ERAD), allowing the translocation of PTS1 from the ER into the cytosol (Banerjee et al. 2016; Pande et al. 2006). This translocation step of PTS1 into the cytosol, occurs with the help of host cell chaperones (Ernst et al. 2018, 2021; Kellner et al. 2019, 2021). Once in the cytosol, PTS1 ADP-ribosylates its target proteins, the α-subunit of inhibitory G-proteins (Gαi) associated with G-protein-coupled receptors (GPCRs) (Bokoch et al. 1983). The modification of Gαi leads to the inhibition of the Gαi-mediated inhibition of the adenylate cyclase (AC), consequently an activation of the AC and an enhanced production of cyclic AMP (cAMP) (Katada and Ui 1982). Therefore, the signal transduction is altered, contributing to specific symptoms associated with the pertussis disease. Earlier, we reported novel strategies to inhibit PT that included antibodies, small molecules, such as chaperone inhibitors, and α-defensin-1 and − 5 (Ernst 2022).

## C2 toxin, anthrax, and diphtheria – uptake, mode of action, and disease pathology

C2 toxin from *Clostridium (C.) botulinum* belongs to the family of binary toxins that are short trip toxins, since their A- and B-subunits are localized on two separate proteins that are also secreted independently from each other. Therefore, the enzymatically active A- and binding/translocation B-subunit of C2 toxin, C2I and C2II respectively (Ohishi 1983a), must assemble in solution or on the cell surface for the formation of the biologically active AB_7_ toxin complexes (Stiles et al. 2011). First, C2II needs to be proteolytically activated into C2IIa which associates into barrel-shaped heptameric complexes that can bind C2I and are taken up into cells through receptor-mediated endocytosis (Barth et al. 2000; Blöcker et al. 2000; Eckhardt et al. 2000). Next, the C2 toxin containing endosomes are acidified which triggers conformational changes of C2IIa that enable the formation of a transmembrane pore within endosomal membranes (Barth et al. 2000). The formed pore mediates the translocation of C2I from the endosomal lumen into the cytoplasm. Here, C2I can execute its enzymatic activity by, mono-ADP-ribosylating G-actin, a key component of the cytoskeleton (Aktories et al. 1986). The modification of G-actin leads to the disruption of the actin cytoskeleton, contributing to the collapse of the cytoskeleton and subsequently to cell rounding and apoptosis of adherent cells (Heine et al. 2008; Wegner and Aktories 1988). In vivo in animals this manifests in a loss of the integrity of the intestinal barrier, causing enterotoxicity (Ohishi 1983b).

The bacterium *B. anthracis* produces two binary toxins, which differ in their enzymatically active A-subunits, lethal factor (LF, a protease) and edema factor (EF, an adenylylcyclase), that are transported by a central B-subunit PA_63_ (protective antigen). PA_63_ binds to target cells and delivers the A-subunit from the endosomes into the cytosol where LF leads to the cleavage of MAP kinases which induces cell death and the severe clinical symptoms of human anthrax (Liu et al. 2024), EF on the other hand leads to edema formation. Inhalation anthrax has a high lethality and was previously abused as bio-terroristic agent, while the combination PA63/LF is a classified biological weapon (Moayeri et al. 2015). Currently, anthrax is treated by the application of antibiotics, while for certain potentially exposed groups, such as the military personal a vaccine is available. Recently, we found the licensed drug, disulfiram to be an inhibitor of the diphtheria toxin, as well as the two binary toxins, C2 toxin and anthrax lethal toxin (Borho et al. 2024).

*Corynebacterium (C.) diphtheriae* produces the single-chain protein toxin, diphtheria toxin (DT), responsible for the highly infectious and vaccine-preventable disease diphtheria which spreads via droplets. DT contains the enzymatically active A-domain (DTA) and the binding/translocation B-domain (DTB). DTB enables DT binding to its receptor, the heparin-binding epidermal growth factor-like growth factor precursor (HB-EGF), on target cells (Naglich et al. 1992). Similarly, to other AB-type toxins DT is internalized into endosomes via receptor-mediated endocytosis, while the translocation of DTA into the cytosol is mediated by a translocation domain located within DTB (Murphy 2011). During the translocation of DTA via the endosomal membranes different host cell factors, such as heat shock protein 90 (Hsp90), protein folding helper enzymes, and thioredoxin reductase are involved (Ratts et al. 2003; Schnell et al. 2015). In the cytosol, DTA executes is enzymatic function as ART and therefore mediates the covalent attachment of ADP-ribose onto a modified histidine residue (diphthamide) of the elongation factor 2 (EF-2), while NAD^+^ serves as a co-substrate. This reaction results in inhibition of protein synthesis and ultimately causes cell death (Collier and Cole 1969). In vivo, the characteristic symptoms of diphtheria include lesions on mucous membranes of pharynx, larynx, tonsils, and nose, that can lead to the obstruction of the airways. Severe complications e.g., myocarditis or nephritis can occur when DT is taken up into the bloodstream (Sharma et al. 2019). Recently, in Western countries diphtheria cases with non-vaccinated children were reported, while in non-vaccinated individuals diphtheria is deadly in up to 10% dure to delayed treatment with antibiotics or DT-neutralizing antiserum (Atkinson et al. 2007; European Centre for Disease Prevention and Control, 2023; European Centre for Disease Prevention and Control., 2025).

## Clostridial toxins TcdA and TcdB–uptake, mode of action, and disease pathology

Toxin A (TcdA) and toxin B (TcdB) are the main virulence factors of the gram-positive, spore-forming, and clinically relevant (nosocomial) bacterial pathogen of the human gut, *Clostridioides (C.) difficile. C. difficile* infections (CDIs) are gastrointestinal infections in humans, leading to mild to severe diarrhea, while severe cases can also lead to life-threatening conditions, including pseudomembranous colitis, colonic perforation, or toxic megacolon, which are also describes as *C. difficile*-associated diseases (CDADs). CDIs are often triggered by a disrupted gut microbiome and majorly contribute to the healthcare burden and high costs. Usually, CDIs develop after antibiotic treatment which creates a favorable environment for *C. difficile* spores in the gut to transform into their vegetative form, to overgrow, and to secrete its toxins.

Those toxins are two highly potent AB-type protein toxins, TcdA (308 kDa) and TcdB (270 kDa), which belong to the family of clostridial glucosylating toxins (CGTs) and are the causative agents of CDI symptoms (Aktories et al. 2017; Voth and Ballard 2005). The large, single-chain proteins with multiple domains, TcdA and TcdB, possess a glucosyltransferase activity. As TcdA/B can be further classified as ABCD-type toxins, they are built up by four functionally different domains. Those domains are responsible for uptake and action within target cells and include the enzymatically active glucosyltransferase domain (GTD) at the N-terminus (A-activity), the cysteine protease domain (CPD) (C-cleavage), the delivery and receptor binding domain (D-delivery), and the CROPs (combined repetitive oligopeptides) domain (B-binding) (Jank and Aktories 2008; Papatheodorou et al. 2024). For both toxins a two-receptor model was postulated, meaning that TcdA/B bind using the CROP domain or the preceding second receptor-binding domain to their specific receptors (Gerhard 2017; Schorch et al. 2014). Receptor binding on target cell surfaces is followed by receptor-mediated and clathrin (TcdB) and/or PACSIN2 (TcdA) endocytosis (Chandrasekaran et al. 2016; Gerhard et al. 2013; Papatheodorou et al. 2010) as well as acidification of endosomes enabled by vesicular adenosine triphosphatases (V-ATPases). This subsequently triggers membrane insertion and pore formation of the toxins (Barth et al. 2001; Orrell et al. 2017), allowing the GTD and the CPD to translocate through the pore into the cytosol (Jank and Aktories 2008). Cytosolic inositol hexakisphosphate (InsP6) binds and activates the CPD for autocatalytic cleavage and release of the GTD into the cytosol (Egerer et al. 2009; Giesemann et al. 2008). The GTD glucosylates small GTPases of the Rho and/or Ras family, including Cdc42, RhoA, and Rac1. This reaction causes the inactivation of the target proteins via the covalent attachment of a glucose moiety from the co-substrate UDP-glucose (mono-O-glucosylation). The GTPases of the Rho family control important cellular functions, including the regulation of the actin cytoskeleton. Consequently, the treatment of cells cultured in a monolayer with TcdA/B leads to the inactivation of GTPases and in the following to a typical morphological feature, the cytopathic cell rounding and ultimately to cell death (Aktories and Just 2005; Hall 1994; Just et al. 1995). In vivo, the inactivation of GTPases disrupts the intestinal barrier which damages the intestine and causes other clinical symptoms that characterize the disease profile of CDIs. Typical symptoms of CDIs are mild to severe diarrhea accompanied e.g., by abdominal pain, fever, vomiting, weakness, and loss of appetite (Czepiel et al. 2019). However, severe cases of CDI can also contribute to life-threatening conditions, including e.g., significant dehydration, pseudomembranous colitis, colonic perforation, and toxic megacolon (Czepiel et al. 2019). The state-of-the-art guideline-recommended treatment of CDIs are antibiotics and include orally applied vancomycin or fidaxomicin as first-line drugs. Patients suffering from multiple recurring episodes of CDI can be also treated using the antibody bezlotoxumab or by a faecal microbiota transplantation (Quan et al. 2024). Recently, our laboratory has shown that the use of licensed drugs, such as amiodarone (Schumacher et al. 2023), domperidone (Braune-Yan et al. 2023), and ambroxol (Heber et al. 2022), can prevent intoxication of cells with TcdA/B (summarized in (Barth et al. 2024).

### Advances of endogenous, antimicrobial, or in silico predicted proteins and peptides as novel inhibitors for toxin-mediated diseases

As outlined above, for many life-threatening infectious diseases that are mediated by the enzymatic activity of highly potent bacterial AB-type protein toxins within target cells, therapeutic options are often limited to antibiotics. Facing increasing resistance of pathogens towards antibiotics, their application is limited, resulting in a treatment gap. Also, preventive measures such as vaccinations become more and more ineffective due to waning immunity and adapting pathogens, leading to escape mutants lacking virulence factors that are part of the vaccination, as it is the case for pertussis (Klein et al. 2012; Ma et al. 2021; Mooi et al. 2014; Tartof et al. 2013; van Boven et al. 2000). Consequently, novel treatment strategies that directly target the causative agents, the bacterial toxins, are urgently required. Ideally, the neutralization or inactivation of the bacterial toxins should prevent intoxication of mammalian target cells and thus inhibit the development of the disease-associated pathology and symptoms.

In the past few years, the field of protein- and peptide-based therapeutics gained importance which is reflected in a continuous increase of approved peptide drugs by the FDA since 2004 (Xiao et al. 2025). Between 2020 and 2023, 21 peptide drugs were approved, while most of the peptide-based drugs in clinical trial phase III are related to diabetes mellitus, followed by COVID-19, and rare diseases (Xiao et al. 2025). Protein- and peptide-based therapeutics are promising drug candidates as small molecules and biologics often lack stability and have a short half-life (Xiao et al. 2025). Peptide therapeutics have several advantages, including lower immunogenicity, better safety profile, higher serum stability, or better distribution within the human body, and lower costs (Xiao et al. 2025).

For the discovery of novel protein- and peptide-based therapeutics, huge potential lies within the human proteome/peptidome (Bosso et al. 2018). The human proteome/peptidome contains more than a million compounds which are often generated using alternative promoters, splicing, and post-translational modifications (Bosso et al. 2018). Additionally, proteolytic processing of precursor proteins/peptides contributes to the enormous set of bioactive compounds, while many of them yet remain unknown (Bosso et al. 2018). Thus, there is a high probability to identify novel bioactive compounds originating from the human body. As those compounds are endogenous, this will also improve the understanding of how the innate immune system fights against bacterial toxins and infections with bacteria. Moreover, this contributes to a better understanding of the disease pathology due to bacterial infections.

**Fig. 3:**
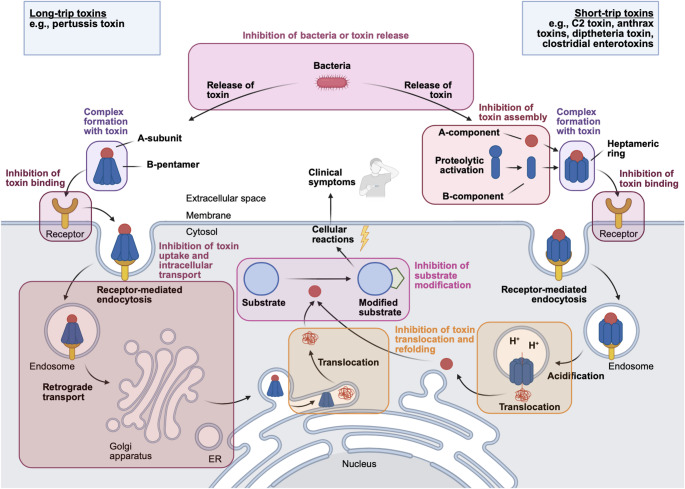
Target structures for inhibition of AB-type protein toxins by endogenous proteins and peptides. Endogenous proteins and peptides can interfere with multiple structures or steps of cellular uptake of bacterial AB-type protein toxins. First, endogenous proteins and peptides can inhibit growth bacteria (e.g., AMPs) and thus release of bacterial toxins. Second, endogenous proteins and peptides can inhibit the assembly of binary AB-type toxins or form complexes with bacterial toxins. Next, the complex formation of proteins/peptides with bacterial toxins might prevent the toxin from receptor binding or proteins/peptides (non)specifically shed target receptors or cell membrane structures required for toxin uptake into cells. Furthermore, intracellular trafficking of bacterial toxins and translocation of the enzyme domain of toxins might be inhibited by proteins/peptides. Lastly, proteins/peptides might inhibit substrate modification and thus prevent further cellular reactions and symptoms of the corresponding toxin-mediated disease. (Created in BioRender. Lietz, S. (2026) https://BioRender.com/qkenkq0)

As highlighted above, AB-type toxins follow a distinct uptake route into target cell to execute their function. Therefore, during the several steps of uptake, there are multiple target structures or processes that could be potentially used for inhibition of AB-type toxins by endogenous proteins and peptides (Fig. 3).

## Search of endogenous proteins and peptides with anti-toxin activity

**Fig. 4:**
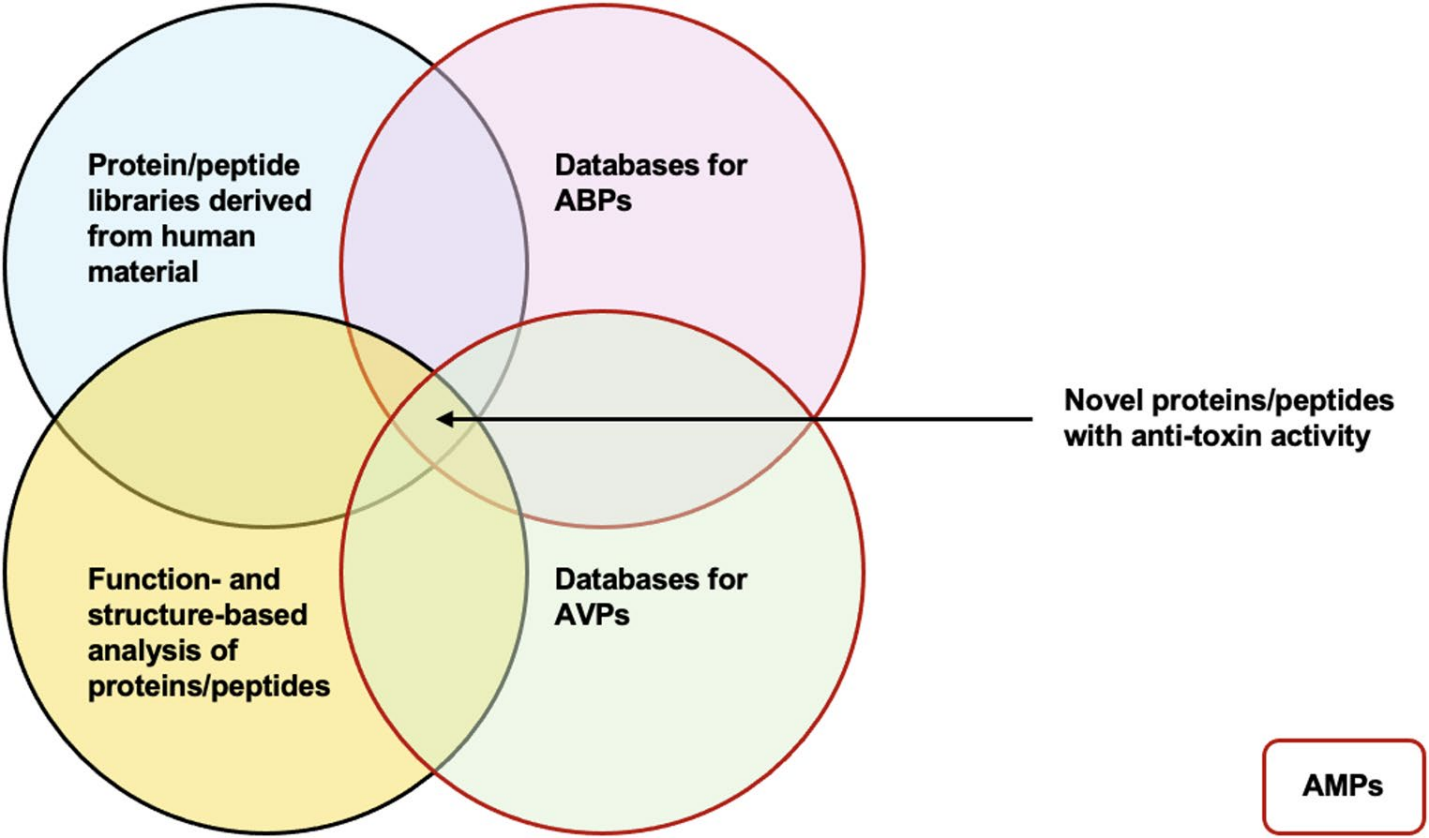
Different strategies can be employed for the identification of novel proteins and peptides with anti-toxin activity. The four different strategies which can be employed for the identification of novel proteins and peptides with anti-toxin activity are screening-based approaches using different starting points. (antimicrobial peptides (AMPs), antibacterial peptides (ABPs), antiviral peptides (AVPs))

For the identification of novel protein and peptide inhibitors of bacterial toxins, different screening-based approaches can be employed (Fig. 4). First, human endogenous protein/peptide libraries originating from different body fluids and tissues can be screened. Second and third, libraries of antimicrobial peptides (AMPs) including antibacterial peptides (ABPs) and antiviral peptides (AVPs) can be analyzed for their anti-toxin activity. Forth, based on a certain function or structure, proteins and peptides can be selected for screening to identify candidates with anti-toxin activity.

## Human protein/peptide libraries as rich sources for toxin-inhibitors

**Fig. 5:**
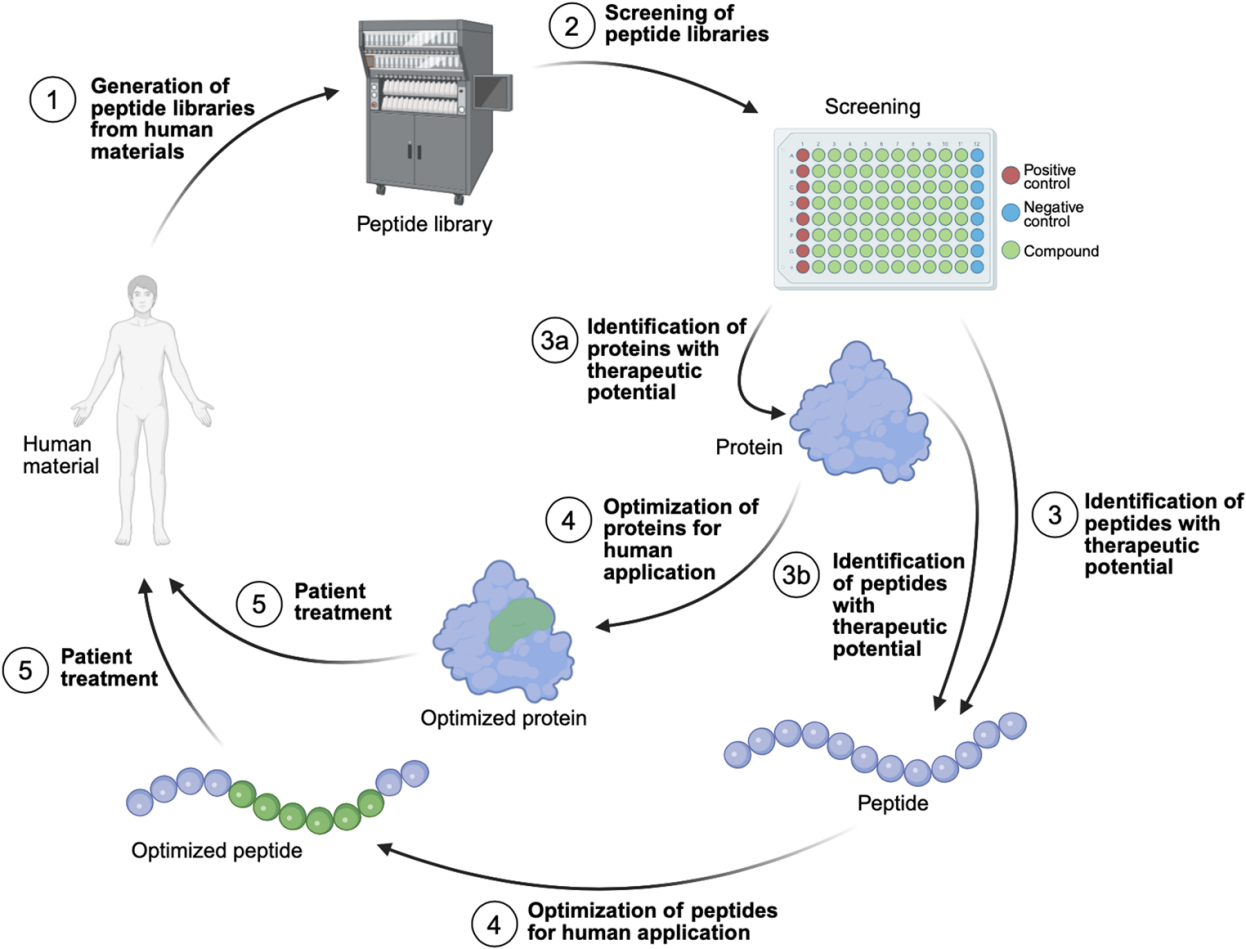
Identification of therapeutic proteins and peptides derived from human materials. First, human materials, including body fluids and tissues from different sources, are collected and processed for the generation of libraries for systematic screenings. The screening procedure is individually designed for the research question/hypothesis. In case of bacterial AB-type protein toxins, this might be an assays analyzing/monitoring for example, the substrate modification (e.g., ADP-ribosylation of Gαi, ADP-ribosylation of actin, or UDP-glucosylation of small GTPases) or subsequent cellular reactions (e.g., cell rounding of cells cultured in a monolayer). The screening procedure ultimately leads to a protein or peptide with inhibitory potential towards one or multiple bacterial toxins. Identified proteins can either directly be used or optimized for human application and patient treatment or can be used as a precursor for the identification of an inhibitory peptide derivative. Inhibitory peptides might be directly used for optimization for human application and patient treatment. (Created in BioRender. Lietz, S. (2025) https://BioRender.com/bb6rrwx)

First, protein/peptide libraries originating from different body fluids and tissues can be screened for anti-toxin activity (Fig. 5). These libraries can be generated from body fluids and tissues, including, e.g., plasma, hemofiltrate, urine, and milk or organs such as, intestines, liver, pancreas, trachea, or lungs. The body fluids and tissues can be obtained from healthy donors or from donors with a certain disease. Tissues and body fluids of infected or diseased donors are particularly interesting as they may have a different composition of proteins and peptides due to ongoing inflammation and tissue degradation or show a specific and characteristic response of the proteome/peptidome towards the pathogen. Therefore, the direct contact to certain pathogens might favor the isolation of antibacterial/antiviral/antitoxin proteins and peptides, as they might be released or produced by (degrading) cells. A more comprehensive overview on the sources of human protein/peptide libraries and their generation would exceed the scope of this article and was summarized earlier (Bosso et al. 2018).

After the generation of human-derived protein/peptide libraries, screening of the libraries takes place (Fig. 5). Here, the selection of an appropriate screening assay is crucial for fast and reliable identification of proteins/peptide with anti-toxin activity. In the following, the requirements for screening assays for the identification of proteins and peptides with anti-toxin activity will be discussed in more detail. After the identification of endogenous toxin inhibitors from human libraries, proteins and peptides can undergo further optimization for human application, in terms of e.g., stability, efficacy, length, charge, hydrophobicity, and toxicity (Rodríguez et al. 2021). Moreover, identified proteins can be further evaluated for the sequence or structure that mediates the anti-toxin activity for subsequent generation of peptides with anti-toxin activity from the protein precursor. These peptides can be subjected to further optimization for human application in a similar manner as mentioned before.

Previously, the screening of human libraries (human hemofiltrate and bronchoalveolar lavage (BAL) was performed successfully for the identification of proteins and peptides with antimicrobial activity, and identified hits were characterized for their mode of virus/bacteria inhibition (Holch et al. 2020; Lawrenz et al. 2025; Münch et al. 2007; Noschka et al. 2021; Wettstein et al. 2021). However, prove of concept for the identification of proteins with anti-toxin activity from human libraries was the identification of α_1_-antitrypsin (α_1_AT) from a human-derived hemofiltrate library as inhibitor for PT from *B. pertussis* (Lietz et al. 2024a, b). Moreover, the systematic screening of peptide derivatives from the human precursor protein α_1_AT lead to the identification of an amino acid sequence region of α_1_AT that possesses anti-PT activity (Lietz et al. 2025a, b). In addition, the unbiased screening of a human hemofiltrate library revealed human angiogenin and its in silico predicted peptide derivative Angie 1 as potent inhibitors against the bacterium *Mycobacterium (M.) tuberculosis* (Noschka et al. 2021). Furthermore, the in silico predicted angiogenin derivatives Angie 3, 5, 6, 7, and reference Angie, including the previously identified AMP Angie 1 were tested for their ability to inhibit bacterial AB-type protein toxins (Lietz et al. 2025a, b). Here, is was shown that Angie 5 is an inhibitor of non-toxin producing *C. difficile*, as well as its secreted toxins TcdA and TcdB (Lietz et al. 2025a, b). This provided further prove of concept that the generation of peptides based endogenous protein precursors can lead to the generation of peptides with anti-toxin activity.

## Antimicrobial peptides and their potential as bacterial toxin inhibitors

Typically, human AMPs result from cleavage of precursor proteins and resemble a defense strategy as part of the innate immunity against invading microorganisms, comprising bacteria, fungi, viruses, and parasites. Characteristically, AMPs have a length of 10–150 amino acids with a L-configuration and are cationic molecules, but the net charge can vary between − 3 and + 20 (Bastos et al. 2017). Often, the inhibitory activity of AMPs is based on their positive charge, hydrophobicity, and amphiphilicity which mostly, results in the disruption of membranes as mechanism of action (Bastos et al. 2017). A more comprehensive overview on AMPs from human body fluids is provided elsewhere (Bastos et al. 2017).

The systematic testing of AMPs might lead to the identification of proteins/peptides with anti-toxin activity. Due to constant exposure of humans to bacteria and viruses, it is likely that the innate immune system might not only has developed strategies to combat bacteria and viruses but also strategies to fight the secreted virulence factors of bacteria, the bacterial toxins. As mentioned in the following, we and others have identified that AMPs have a huge potential to be toxin inhibitors.

### Antibacterial peptides and their potential as bacterial toxin inhibitors

Upon bacterial infection humans are exposed to the bacteria and its endo- and exotoxins. Endotoxins are mostly released during bacterial lysis and comprise e.g., parts of cellular membranes including lipopolysaccharides (LPS). In contrast, exotoxins are secreted virulence factors e.g., AB-type protein toxins. Since the AB-type protein toxins are the causative agents for the symptoms of the disease manifesting after bacterial infection, it is likely that the innate immune system also developed strategies to combat bacterial toxins. There is the possibility that inhibitory properties of ABPs might be also required or shared with peptides that inhibit bacterial AB-type protein toxins e.g., hydrophobicity or charge.

Human defensins are endogenous, cationic peptides with a β-sheet structure and three intramolecular disulfide bridges (Ganz 2003; Kim and Kaufmann 2006). They can be classified according to their cysteine pairing and their structure into three groups, α-, β-, and θ-defensins (Ganz 2003; Kim and Kaufmann 2006). Human defensins have antibacterial activity against a broad spectrum of gram-negative and -positive bacteria and their predominant mode of action is the disruption of bacterial membranes and the inhibition of cell wall synthesis, while outer membrane proteins, LPS, peptidoglycan, lipid II, phosphatidylglycerol, and lipoteichoic acids, as well as bacterial DNA and RNA are primarily bacterial targets of human defensins (Nagib et al. 2025). Interestingly, human defensins also have antiviral activity, e.g., against SARS-CoV-2 (Nagib et al. 2025; Solanki et al. 2021). However, aside the antimicrobial activity, human defensins, predominantly the α-defensins, were shown to possess activity against multiple AB-type protein toxins, including iota toxin, CDT, TcdA, and TcdB, DT, anthrax LT, and PT (Barthold et al. 2022; Fischer et al. 2018, 2020; Giesemann et al. 2008; Kim et al. 2005, 2006; Kling et al. 2021, 2023; Korbmacher et al. 2020). The mechanism of action of the defensins is often related to the aggregate formation with or binding to bacterial AB-type toxins or inhibition of enzyme activity and will be explained in more detail in the following. Next to the human defensins, as mentioned earlier, the in silico predicted Angie peptides derived from human angiogenin and angiogenin itself bear antibacterial activity (Lietz et al. 2025a, b; Noschka et al. 2021). At the same time the Angie peptides also showed activity against TcdA and TcdB (Lietz et al. 2025a, b). This underlines the huge potential of proteins/peptides with antibacterial activity to also act against bacterial toxins.

## Antiviral peptides and their potential as bacterial toxin inhibitors

**Fig. 6:**
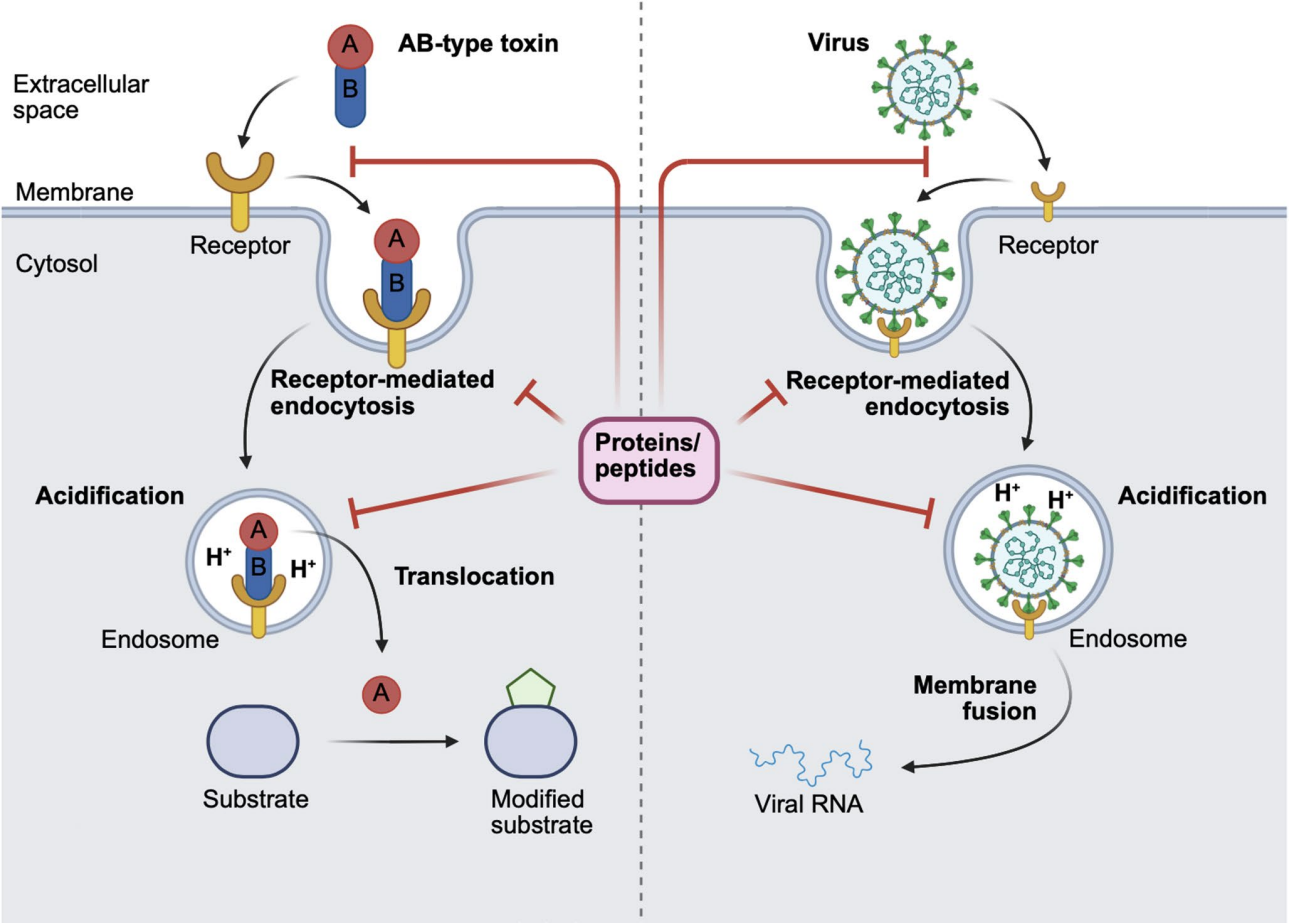
Bacterial AB-type toxin uptake vs. virus uptake. The early steps of bacterial AB-type toxin (left) and virus (right) uptake are comparable. After binding to cell surface receptors, both, bacterial AB-type toxins (short-trip toxins, e.g., C2 toxin) and viruses (e.g., SARS-CoV-2), are taken up via receptor-mediated endocytosis into endosomes. Next, the endosomes are acidified which in case of bacterial AB-type toxins triggers the translocation of the enzymatically active A-component into the cytosol. Here, the A-component modifies its corresponding substrate. Comparably, in case of viruses the acidification of endosomes triggers the membrane fusion of virus and endosome which enables the release of genetic material of viruses, e.g., viral RNA. (Created in BioRender. Lietz, S. (2025) https://BioRender.com/opgrg7q)

Systematic testing of peptides with antiviral activity might lead to the identification of peptides with anti-toxin activity, since some viruses share an uptake route which is comparable to the uptake of bacterial AB-type protein toxins (Fig. 6). For example, after binding to the angiotensin-converting enzyme 2 (ACE2) receptor, SARS-CoV-2 viruses can be taken up into mammalian target cells through receptor-mediated endocytosis (Jackson et al. 2022). Next, the endosomes comprising the virus particles are acidified which enables the membrane fusion of the virus and the endosome, causing the release of viral RNA into the cytosol (Jackson et al. 2022). Therefore, especially, the steps of receptor-mediated endocytosis and acidification of endosomes are shared between some viruses and bacterial AB-type toxins (Fig. 6). Consequently, proteins/peptides that inhibit e.g., receptor-mediated endocytosis or acidification of endosomes might inhibit the uptake of bacterial AB-type toxins and viruses in a similar fashion.

For example, the FDA-approved malaria drug chloroquine and its derivative hydroxychloroquine, inhibit SARS-CoV-2 entry via endosomal pathway in vitro into cells (Jackson et al. 2022; Yuan et al. 2022). In detail, chloroquine and hydroxychloroquine lead to an increased endosomal pH of cells and showed inhibition of virus that are dependent on the acidification of endosomes for entry into cells (Hoffmann et al. 2020; Rolain et al. 2007). However, clinical studies involving chloroquine and hydroxychloroquine for COVID-19 treatment showed no benefit and safety issues involving cardiac side effects (FDA, 2025; Gasmi et al. 2021). Anyhow, it was also shown that the positively charged heterocyclic molecules, chloroquine and derivatives e.g., quinacrine inhibit also bacterial AB-type proteins, C2 toxin, iota toxin, and anthrax lethal toxin (Bachmeyer et al. 2001, 2003; Benz and Barth 2017; Kreidler et al. 2017; Kronhardt et al. 2016; Neumeyer et al. 2008; Orlik et al. 2005; Schmid et al. 1994). Here, chloroquine and derivatives showed an interaction with the B-component C2IIa and PA_63_ of C2 toxin and anthrax lethal toxin respectively and thus functioned as a pore blocker (Benz and Barth 2017). These findings led to the assumption that the inhibitor binding is enabled by the ion-ion interactions of the positively charged inhibitors with the negatively charged vestibule of the channels of B-components (Benz and Barth 2017).

Moreover, it was previously shown that antiviral proteins and peptides can also be identified from human endogenous protein/peptide libraries, e.g., the identification of α_1_AT from a human BAL library as SARS-CoV-2 inhibitor (Wettstein et al. 2021) or the α_1_AT derived peptide VIRIP from human hemofiltrate that inhibits HIV-1 (Münch et al. 2007). Despite the different mode of action, α_1_AT was also identified from human hemofiltrate as a very potent multi-toxin inhibitor for PT, C2 toxin, DT, and an anthrax fusion toxin (Lietz et al. 2024a, b; Lietz et al. 2024a, b). Moreover, a short sequence of human endogenous α_1_AT was also identified to have anti-PT activity, while VIRIP showed no inhibition (Lietz et al. 2025a, b).

## Structure- and function-based selection of proteins/peptides for screening of toxin inhibitors

**Fig. 7:**
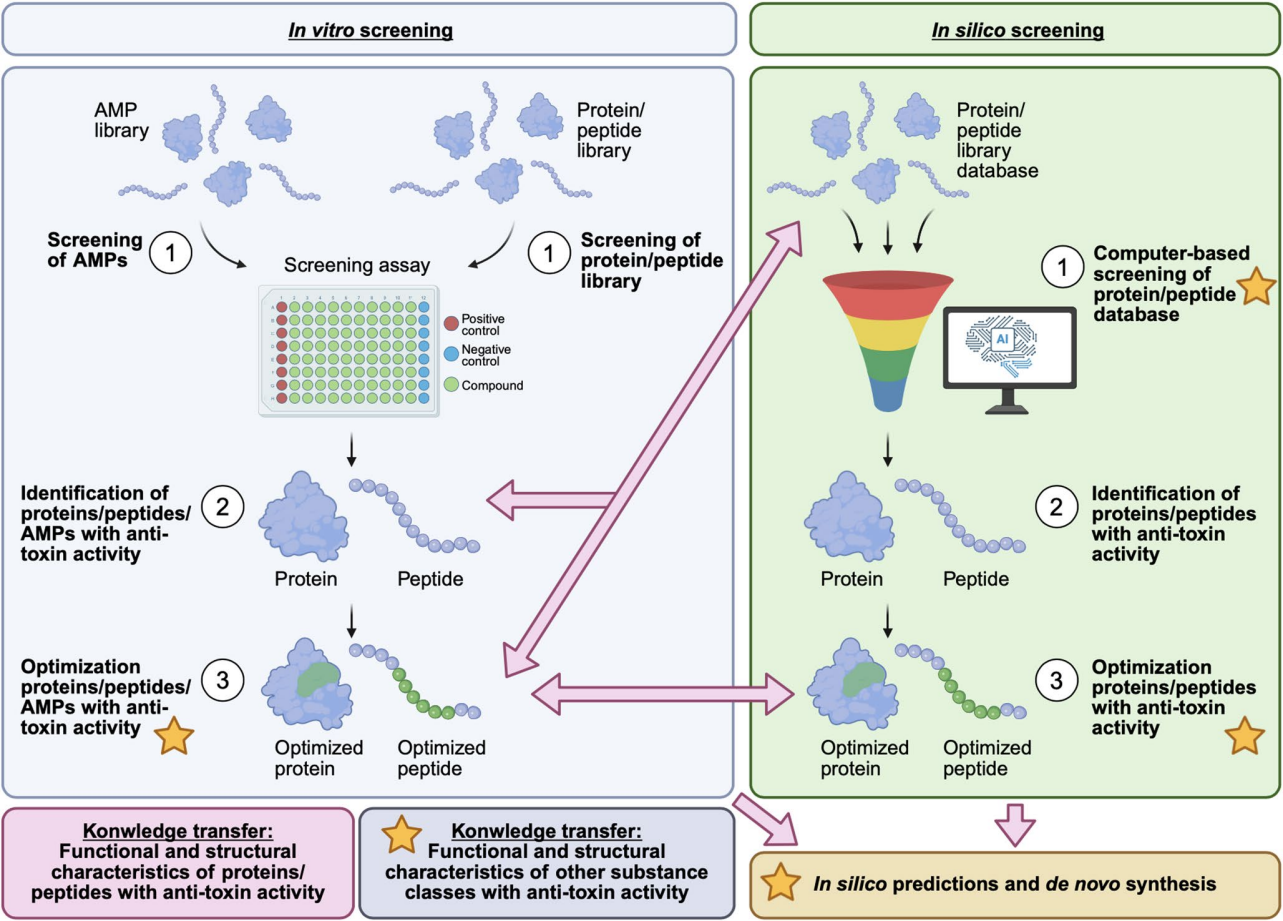
Structure- and function-based selection of proteins/peptides for screening of toxin inhibitors. In vitro and in vivo screening approaches to identify proteins/peptides with anti-toxin activity can complement each other. Protein/peptide libraries from e.g., human materials or libraries of AMPs can be screened in biological assays for anti-toxin activity. After identification and optimization of proteins/peptides with anti-toxin activity, common characteristics of the toxin inhibitors can be identified. In a similar manner, databases of proteins/peptides can be screened according to those previously identified characteristics to identify and optimize novel proteins/peptides with anti-toxin activity. Identified and isolated proteins/peptides from in vitro and in silico screenings need to be tested for their anti-toxin activity in biological assays, as well as after their optimization. Moreover, there is a knowledge transfer between in vitro and in vivo screenings. Identified characteristics from in vitro screenings can be used during in silico screenings but in vitro screening results could be checked for certain characteristics from anti-toxin databases. In addition, during optimization of proteins/peptides with anti-toxin activity there is a knowledge transfer as in vitro results can improve in silico predictions for optimization and predicted improvements could results in higher anti-toxin activity. Moreover, during in silico screening and during optimization of peptides knowledge on functional and structural characteristics of other substance classes such as small molecules with anti-toxin activity could be beneficial. In addition, in vitro and in vivo screening results could be used for the in silico prediction and *de novo* synthesis of peptides. (Antimicrobial peptide (AMP)) (Created in BioRender. Lietz, S. (2026) https://BioRender.com/hkhyjsb)

In general, two screening approaches can be employed for the identification of novel protein/peptide-based inhibitors with anti-toxin activity, in vitro and in silico screenings (Fig. 7). However, these two screening approaches are interconnected due to the transfer of experimental and computational gained knowledge and thus complement each other.

First, in vitro screenings that aim to identify bacterial toxin inhibitors can be conducted using any library e.g., protein/peptide libraries originating from human materials or libraries of proteins/peptides with antimicrobial activity. After the identification of proteins/peptides with anti-toxin activity, they can be further optimized using in silico tools. Second, in silico screenings can be performed using collected databases of protein/peptide libraries. These databases might be based on existing proteins/peptide libraries from e.g., human materials which were analyzed for their composition. The identified hits of proteins/peptides can be matched with information collected from literature and therefore might comprise different information. This information of proteins/peptides could be e.g., biological function, interaction partners, length, size, charge, 3D structure, and hydrophobicity but also other characteristics depending on the aim of the screening. Based on certain characteristics which were previously selected, e.g., due to experimental data collected from in vitro screenings, in silico screenings are conducted where proteins/peptides can be ranked for their potential inhibitory activity towards bacterial toxins. As in silico screening results are predicted results, they require experimental validation of top ranked hits. Consequently, biological (screening) assays are employed for identification and optimization of anti-toxin protein/peptide inhibitors during in vitro screenings but also for the experimental validation of predicted and optimized inhibitors through in silico screenings.

Since in vitro and in silico screenings can complement each other, there is also a transfer of knowledge that can be used to improve subsequent screenings. For example, after the identification and optimization of proteins/peptides derived from in vitro screenings, knowledge on certain characteristics e.g., structure or function of bacterial toxin inhibitors can be collected. This information can be used for the screening of protein/peptide databases for the identification of novel inhibitors. Moreover, protein/peptide databases could already comprise modified versions of (optimized) proteins/peptides that are eligible for in vitro testing. Next, the knowledge transfer during the optimization of proteins/peptides during in vitro and in vivo screenings can occur in both directions. The experimental data from in vitro tests of optimized protein/peptides can help to improve the in silico optimization of potential anti-toxin inhibitors. However, also in silico optimizations can result in knowledge on structural and functional characteristics of protein/peptide inhibitors for bacterial toxins that can be transferred and applied during the optimization of in vitro screening hits. Furthermore, in vitro and in silico screenings generally improve in silico prediction tools for identifying and optimizing proteins/peptides with anti-toxin activity. This might also lead to the in silico prediction and *de novo* synthesis of novel not yet identified peptides with anti-toxin activity. For example, the Monte Carlo-based peptide binding design (PepBD) algorithm and explicit-solvent atomistic molecular dynamics simulation were used for the prediction of peptide-based inhibitors targeting the catalytic site (GTD) of TcdA, while the in silico predicted peptide inhibitors were subsequently validated experimentally (Xiao et al. 2022).

Last, there is a knowledge transfer on structure and function of anti-toxin inhibitors of substance classes that are not proteins/peptides such as small molecules. Here, e.g., small molecules that inhibit the endosomal uptake route including acidification of endosomes or molecules that function as pore blockers could possibly inhibit the uptake of bacterial AB-type protein toxins into target cells. Therefore, literature research could contribute to the generation of proteins/peptides (or other molecules) libraries with certain function for systematic in vitro and in silico screening.

Characteristics that might be important for the identification and optimization of proteins/peptides with anti-toxin activity include e.g., structural characteristics, function, length, charge, hydrophobicity. As already mentioned, these characteristics are also important for the activity of AMPs, ABPs and AVPs (Neghabi Hajigha et al. 2024). Consequently, the identification of proteins/peptides with antimicrobial activity might lead to the identification of proteins/peptides with antitoxin activity and vice versa.

Important structural characteristics can be related to the amino acid composition either by their sequence or the presence of hydrophobic and cationic residues or reactive amino acids i.e., cysteine residues (Neghabi Hajigha et al. 2024). Moreover, also the secondary structure and the resulting 3D structure could play an important role for the e.g., the sterically interaction with the enzyme domain of the bacterial toxins for enzymatic inactivation or the blocking of pores formed by the B-component of binary AB-type toxins. In a similar manner length, charge, and hydrophobicity could play a role for mediating the inhibitory effect which is mediated by the direct interaction of proteins/peptides and bacterial toxins through aggregate formation. Furthermore, the protein/peptide could possess and physiological function e.g., as protease inhibitor. Since some bacterial AB-type protein toxins, C2 toxin, DT, and anthrax toxin, are dependent on trypsin or furin cleavage for activation, proteolytical cleavage of trypsin or furin would inhibit the toxins.

Others already summarized characteristics of the group of human defensins that are correlated to activity of defensins and inhibition of bacterial toxins (Kudryashova et al. 2017). Important characteristics of defensins are the especially related to their structure including the cysteine residues that form the stabilizing disulfide bonds, β-bulge, salt bridges, and chirality (Giesemann et al. 2008; Kim et al. 2005; Kling et al. 2023; Kudryashova et al. 2014, 2015, 2017; Lehrer et al. 2009; Wei et al. 2009). Moreover, hydrophobicity, dimerization, and charge of the defensins are important characteristics (Kudryashova et al. 2017). Therefore, these characteristics might also be crucial for the antitoxin activity of other proteins/peptides. For example, the angiogenin derived Angie peptides with a positive Gravy (Grand average of hydropathicity) values showed inhibition of TcdA and TcdB, while Angie peptides with a negative Gravy score showed no inhibition (Lietz et al. 2025a, b). Additionally, also for the heptamer PA_63_ of anthrax toxin hydrophobic peptide sequences were identified to inhibit toxin formation (Glick et al. 2002; Gujraty et al. 2005; Mourez et al. 2001) and for shiga toxin potential therapeutic peptides were identified where in silico modeling revealed the importance of hydrophobic interactions between the toxin and the peptide inhibitor (Li et al. 2016). Consequently, the hydrophobicity of proteins/peptides seems to play a role during inhibition of TcdA and TcdB but also for other bacterial AB-type protein toxins.

Finally, identified protein/peptides with antitoxin activity could be further optimized, while considering above listed characteristics. For example, alanine mutagenesis can help to identify amino acid residues that are important for mediating the antitoxin effect as performed for the endogenous PT inhibitor α_1_AT (Lietz et al. 2025a, b). Consequently, residues that are not relevant could be exchanged with residues that effect overall charge or hydrophobicity of the protein/peptide to improve its antitoxin activity. In a similar manner, truncated peptides can help to optimize the protein/peptide length and identify core amino acids that mediate the antitoxin effect. In silico tools for modeling the interaction between protein/peptide and the toxin (Kling et al. 2023; Lietz et al. 2024a, b, 2025a, b) can assist during the optimization of the mode of action. Lastly, modifications that enhance stability of the protein/peptide and reduce toxicity are crucial, especially for later applications in vivo.

### Screening assays for the identification of proteins and peptides with anti-toxin activity

The systematic screening of protein/peptide libraries which may contain hundreds of samples with a unique protein/peptide mixture is crucial for the identification of proteins and peptides with anti-toxin activity. Therefore, it is essential that the screening assay meets certain key criteria and addresses the research question. In general, the ideal screening assay is a high-throughput assay which is easy to process, fast, and cost-effective. In addition, the validation of the screening assay with appropriate controls that also should be included into the routine screening of the protein/peptide libraries is of importance. The analyzed endpoint of the screening assay should be robust and reliable. For example, if it is required that several cell lines or cell systems should be analyzed at once, it would be advantageous that the endpoint evaluated is the same or at least comparable in all systems for an easier evaluation of the results. Finally, the screening assay should identify proteins and peptides with anti-toxin activity with high specificity and sensitivity. However, often compromises need to be made between the listed criteria when a screening assay is selected.

The screening assay which lead to the identification of α_1_AT as inhibitor for PT from *B. pertussis* was a Western Blot based assay where the analyzed endpoint was the ADP-ribosylation of Gαi in PT-treated CHO-K1 cells (Lietz et al. 2024a, b). Despite Western Blot is a rather time-consuming method, this assay was selected, since it allowed the reliable detection of the ADP-ribosylation of Gαi by PTS1. The enzyme reaction where PTS1 ADP-ribosylates Gαi represents an endpoint analysis of PT-mediated effects which is rather downstream. Meaning inhibitory effects on PT upstream of the ADP-ribosylation of Gαi including the uptake and intracellular trafficking of PT are captured when analyzing the ADP-ribosylation status of Gαi. Therefore, when analyzing the substrate modification and subsequent cellular reactions as readout, many potential inhibitors with different modes of inhibition are included. Since many bacterial toxins lead to the collapse of the actin cytoskeleton and subsequently to cell death, screening assays could also monitor cell morphology via light microscopy. This screening assay would be easy and fast to perform, while the readout is very robust.

However, it might be desired to identify inhibitors with a certain mode of inhibition, e.g., the inhibition of toxin binding to receptor or other target structures on cell surfaces facilitating toxin uptake such as sugars. In this case, the screening assay could be a high through-put flow cytometry-based assay that monitors binding of a fluorescently labeled toxin to cells. In a similar fashion screening assays could be designed that analyze the intracellular trafficking of the toxins or the translocation of the toxin from cellular compartments (e.g., the ER or endosomes) into the cytosol. Lastly, the enzyme activity of toxins can also be investigated in the presence of the potential inhibitor in cell-free systems, by mixing enzyme, substrate, inhibitors, and required co-factors in buffer. Such a cell-free assay might be advantageous since they reduce unpredictable effects due to cellular components and have a reduced complexity due to defined components. On the other hand, screenings performed in cell-free assays might not represent physiological conditions due to the reduced complexity. However, in general FCS-free conditions during screening are beneficial to exclude effects of proteins within FCS or between different FCS charges.

### Examples of endogenous proteins of peptides with anti-toxin activity

In the following, examples of human proteins and peptides or thereof derived peptide derivatives are given. Subsequently, single proteins and peptides were selected, and their mode of toxin inhibition are described in more detail (Table 1).

**Table 1:** Examples for human endogenous proteins and peptides with anti-toxin activity. Human derived proteins and peptides that inhibit bacterial toxins listed with the respective mode of action. (Kudryashova et al. 2017)

Human serum albumin (HSA)				
Toxin	Bacterium	Inhibitor	Mechanism of inhibition	References
TcdA	*C. difficile*	HSA	Toxin binding and induction of autoproteolytic cleavage	(di Masi et al. 2018)
TcdB	*C. difficile*	HSA	Toxin binding and induction of autoproteolytic cleavage	(di Masi et al. 2018)

Human α- and β-defensins (def)				
Toxin	Bacterium	Inhibitor	Mechanism of inhibition	References
PT	*B. pertussis*	α-def-1, α-def-2, α-def-3, α-def-4, α-def-5	Inhibition of enzyme activity and inhibition of toxin binding to cells Toxin inhibition, mechanism not tested yet Toxin inhibition, mechanism not tested yet Toxin inhibition, mechanism not tested yet Inhibition of enzyme activity and inhibition of toxin binding to cells	(Kling et al. 2021, 2023)
PT	*B. pertussis*	α-def-6, β-def-1, β-def-2	No inhibition No inhibition No inhibition	(Kling et al. 2021, 2023)
Iota toxin	*C. perfringens*	α-def-1	Interaction with Ib	(Fischer et al. 2018)
Iota toxin	*C. perfringens*	β-def-1	No inhibition	(Fischer et al. 2018)
LF	*B. anthracis*	α-def-1, α-def-1-3, α-def-5	Inhibition of enzyme activity and aggregate formation Inhibition of enzyme activity Aggregate formation	(Giesemann et al. 2008; Kim et al. 2005; Wei et al. 2009)
PA	*B. anthracis*	α-def-1, α-def-5	Aggregate formation Aggregate formation	(Giesemann et al. 2008)
LF	*B. anthracis*	β-def-3	Inhibition of enzyme activity	(Wei et al. 2009)
LT	*C. sordellii*	α-def-1, α-def-5	No aggregate formation No aggregate formation	(Giesemann et al. 2008)
DT	*C. diphtheriae*	α-def-1-3	Inhibition of enzyme activity	(Kim et al. 2006)
TcdA	*C. difficile*	α-def-1, α-def-5, α-def-6	Toxin binding and aggregate formation Aggregate formation Toxin inhibition, mechanism not tested yet	(Barthold et al. 2022; Fischer et al. 2020; Korbmacher et al. 2020)
TcdB	*C. difficile*	α-def-1, α-def-3, α-def-5, α-def-6	Toxin binding, aggregate formation, and inhibition of enzyme activity Aggregate formation and inhibition of enzyme activity Aggregate formation and inhibition of enzyme activity Toxin binding, aggregate formation	(Barthold et al. 2022; Fischer et al. 2020; Giesemann et al. 2008; Korbmacher et al. 2020)
TcdA	*C. difficile*	β-def-1	No inhibition	(Fischer et al. 2020)
TcdB	*C. difficile*	β-def-1	No inhibition	(Giesemann et al. 2008)
CDT	*C. difficile*	α-def-1, α-def-5	Toxin binding Inhibition of CDTb	(Barthold et al. 2022; Fischer et al. 2020; Korbmacher et al. 2020)
Exotoxin A (ETA)	*Pseudomonas*	α-def-1-3	Inhibition of enzyme activity	(Kim et al. 2006)
gNarE	*N. gonorrhoeae*	β-def-1, β-def-2, β-def-4	Inhibition of enzyme activity Inhibition of enzyme activity Inhibition of enzyme activity	(Rodas et al. 2016)
Panton-Valentine leukocidin (PVL)	*S. aureus*	α-def-3	Inhibition of pore formation	(Cardot-Martin et al. 2015)
PVL	*S. aureus*	α-def-1, α-def-2	No inhibition No inhibition	(Cardot-Martin et al. 2015)
MARTX	*V. cholerae*, *A. hydrophila*	α-def-1, α-def-5	Inhibition of enzyme activity of actin crosslinking domain and CPD	(Kudryashova et al. 2014)
Cholesterol-dependent cytolysins (CDCs)		α-def-1, α-def-2, α-def-3, α-def-4, α-def-5, α-def-6	Inhibition of hemolysis Inhibition of hemolysis Inhibition of hemolysis No inhibition Inhibition of hemolysis with lower potency No inhibition	(Lehrer et al. 2009)
CDCs		β-def-1, β-def-2, β-def-3	No inhibition No inhibition No inhibition	(Lehrer et al. 2009)

Human α_1_-antitrypsin (α_1_AT)				
Toxin	Bacterium	Inhibitor	Mechanism of inhibition	References
PT	*B. pertussis*	α_1_AT	Inhibition of binding	(Lietz et al. 2024a, b)
PT	*B. pertussis*	Antithrombin, antithrombin with fondaparinux	No inhibition	(Lietz et al. 2024a, b)
PT	*B. pertussis*	α_1_AT-HF, α_1_AT-HF P8, 42, 64, (α_1_AT-derived peptides)	Inhibition of intoxication, mechanism not tested yet	(Lietz et al. 2025a, b)
PT	*B. pertussis*	VIRIP, and other α_1_AT-derived peptides	No inhibition	(Lietz et al. 2025a, b)
C2 toxin	*C. botulinum*	α_1_AT	Inhibition of C2 toxin binding and inhibition of C2I enzyme activity	(Lietz et al. 2024a, b)
C2 toxin	*C. botulinum*	α_1_AT-HF (α_1_AT-derived peptide)	Inhibition of intoxication, mechanism not tested yet	(Lietz et al. 2025a, b)
Anthrax fusion toxin [PA_63_]_7_ + LF_N_DTA	*B. anthracis*	α_1_AT	Inhibition of heptameric binding subunit PA63	(Lietz et al. 2024a, b)
DT	*C. diphtheriae*	α_1_AT	Inhibition of intoxication, mechanism not tested yet	(Lietz et al. 2024a, b)
TcdA	*C. difficile*	α_1_AT	No inhibition	(Lietz et al. 2024a, b)
TcdB	*C. difficile*	α_1_AT	No inhibition	(Lietz et al. 2024a, b)
CDT	*C. difficile*	α_1_AT	No inhibition	(Lietz et al. 2024a, b)

Toxin	Bacterium	Inhibitor	Mechanism of inhibition	References
LF + [PA_63_]_7_	*B. anthracis*	Inter-alpha-inhibitor protein (IαIp) (endogenous serin protease inhibitor)	Inhibition of furin which cleaves PA_83_ for activation and formation of LT	(Opal et al. 2005)

In silico predicted peptides derived from human angiogenin (Angie peptides)				
Toxin	Bacterium	Inhibitor	Mechanism of inhibition	References
TcdA	*C. difficile*	Angie 1, 3, and 5	Inhibition of intoxication, mechanism not tested yet	(Lietz et al. 2025a, b)
TcdA	*C. difficile*	Angie 6, 7, and reference Angie	No inhibition	(Lietz et al. 2025a, b)
TcdB	*C. difficile*	Angie 1, 3, and 5	Inhibition of intoxication, mechanism not tested yet	(Lietz et al. 2025a, b)
TcdB	*C. difficile*	Angie 6, 7, and reference Angie	No inhibition	(Lietz et al. 2025a, b)

### Human serum albumin as inhibitor for TcdA and TcdB

As the most abundant protein in the human plasma HSA acts as a physiological buffer. Severe CDIs, the recurrent, and fatal diseases are associated with low HSA levels (Bella et al. 2015; di Masi et al. 2018; Kumarappa et al. 2014; Tabak et al. 2015; Walker et al. 2013). To investigate the role of HSA as inhibitor for TcdA and TcdB, a combined approach of in silico, in vitro and in vivo experiments was employed. Using in silico docking simulations and biochemical analyses executed in vitro on purified proteins and different models, including human epithelial colorectal adenocarcinoma cells (Caco-2) and in vivo on stem cell-derived human intestinal organoids, and zebrafish embryos, the function of HSA was characterized as part of host defense mechanism against *C. difficile* infection (di Masi et al. 2018; Tonon et al. 2020). When CaCo-2 cells were intoxicated with TcdB or TcdB and treated with HSA, an increased cell viability was observed. Therefore, HSA protected cells from intoxication with TcdB or TcdB and TcdA and additionally preserved the epithelial integrity. Moreover, in more complex models involving human induced pluripotent stem cell-derived intestinal organoids and in zebrafish embryos, HSA protected intestinal crypts of the organoids from intoxication with TcdA and TcdB and protected zebrafish embryos from TcdB-mediated effects (di Masi et al. 2018; Tonon et al. 2020). Furthermore, the results demonstrated that at physiological concentrations the autoproteolytic cleavage of TcdA and TcdB was induced through the predicted and specific binding of the hydrophobic portions of the DD domains of TcdA and TcdB with the domain II of HSA. The toxin binding outside of cells consequently, inhibited toxin internalization and subsequent steps of the toxin-dependent glucosylation of Rho proteins in CaCo-2 cells. This revealed that HSA is an essential component of the host defense mechanism against *C. difficile* intoxication which simultaneously provides evidence for the clinical correlation between CDI severity and hypoalbuminemia.

### Group of defensins as inhibitors for iota toxin, TcdA, TcdB, CDT, and PT

Human defensins are endogenous AMPs and can be classified according to their structure into two groups, α- and β-defensins (Kim and Kaufmann 2006). Several α-defensins, including α-defensin-1, -5, and − 6 were identified to protect cells in vitro and in vivo from the cytotoxic effects mediated by a set of short- and long-trip AB-type toxins, including iota toxin (Fischer et al. 2018), the clostridial toxins CDT, TcdA, and TcdB (Barthold et al. 2022; Fischer et al. 2020; Giesemann et al. 2008; Korbmacher et al. 2020), and PT (Kling et al. 2021, 2023).

*Clostridium (C.) perfringens* produced iota toxin was inhibited in cells-based assays using human colon cells when α-defensin-1 was added. In contrast, β-defensin-1 was not able to protect cells from intoxication with iota toxin which indicated the specificity of the mode of action for the α-defensin-1 (Fischer et al. 2018). For the inhibition of iota toxin mediated by α-defensin-1, Ib needed to be preincubated on cells before the addition of Ia. Moreover, in absence of Ia α-defensin-1 was able to protect cells from Ib-mediated effects which indicates that α-defensin-1 most likely interacts with Ib and interferes with the formation of the biologically active iota toxin. Moreover, α-defensin-1 had no effect on binding of iota toxin to cells and on the enzyme activity of Ia when analyzing the ADP-ribosylation of actin in vitro. This highlights α-defensin-1 as promising endogenous peptide candidate for the treatment of diseases associated with iota toxin.

In addition to iota toxin, α-defensin-1 inhibited TcdA, TcdB, and CDT from *C. difficile* in different cell-based assays analyzing the cell morphology, substrate modification, and transepithelial electrical resistance (Fischer et al. 2020; Giesemann et al. 2008). In cell morphology-based assays, α-defensin-1 protected Vero and CaCo-2 cells from cytopathic effects of TcdA, TcdB, and the combination of both toxins, while in CaCo-2 TcdA, TcdB, and TcdA and TcdB-mediated glucosylation of Rac-1 was inhibited by α-defensin-1. In the same assay, β-defensin-1 did not inhibit TcdA-mediated glucosylation of Rac-1 in of CaCo-2 cells. In vitro, the enzyme activity of TcdB was inhibited by α-defensin-1, -3, and − 5 (Giesemann et al. 2008). Moreover, α-defensin-1 protected the integrity of the epithelial barrier function of a CaCo-2 cell monolayer treated with TcdA. In a comparable manner α-defensin-1 inhibited intoxication of CaCo-2 cells with the binary toxin CDT and when all three toxins, TcdA, TcdB, and CDT were combined. In addition, also cells arranged in a more sophisticated model, a human intestinal organoid model was protected from the combined effects of TcdA, TcdB, and CDT when treated with α-defensin-1. The molecular mode of inhibition mediated by α-defensin-1 is based on the direct interaction of TcdA, TcdB, CDTb with α-defensin-1 in vitro. Furthermore, incubation of α-defensin-1 with TcdA led to aggregate formation as well as α-defensin-1, -3, and − 5 with TcdB(Fischer et al. 2020; Giesemann et al. 2008). In vivo, in mice the TcdA-induced damage of intestinal loops was reduced due to α-defensin-1.

Apart from α-defensin-1, α-defensin-5 was also shown to inhibit TcdA, TcdB, and CDT, as well as the medically relevant combination in human-derived cell lines (Giesemann et al. 2008; Korbmacher et al. 2020). In a similar fashion as for α-defensin-1, the effects of α-defensin-5 were further investigated in assays analyzing toxins-induced changes in cell morphology, intracellular substrate modification, and decrease of trans-epithelial electrical resistance (TEER). In cell-based experiments, α-defensin-5 showed a time- and concentration-dependent delay of intoxication of cells with the combination of TcdA, TcdB, and CDT. In vitro, the ezmye activity of TcdA and CDTa was not inhibited by α-defensin-5. The mode of inhibition mediated by α-defensin-5 most likely relied on the aggregate formation of of α-defensin-5 with TcdA causing the inactivation of TcdA (Korbmacher et al. 2020). The inhibition of CDT though α-defensin-5 might be mediated by inhibition of the formation of cytotoxic pores by CDTb (Korbmacher et al. 2020).

Next, α-defensin-6 was shown to inhibit TcdA, TcdB, and the combination of both toxins in vitro in human cells and a model of epithelial barriers (Barthold et al. 2022). Comparable to α-defensin-5, α-defensin-6 was shown to bind and form complexes with TcdB, that mediates the inactivation of TcdB, preventing cytotoxic activity. The enzyme activity of TcdB and the autoproteolytic processing was not inhibited by α-defensin-6. Moreover, in vivo, α-defensin-6 protected zebrafish embryos from intoxication with TcdB.

Consequently, α-defensin-1, -5, and − 6 are promising peptide inhibitors of the clostridial toxins, TcdA, TcdB, and CDT and could be used as treatment strategies for CDIs.

Interestingly, human α-defensins did not only show inhibition of the short trip toxins, iota toxin, TcdA, TcdB, and CDT, but also inhibited the long-trip toxin PT (Kling et al. 2021, 2023). Here, α-defensin-1 and − 5 significantly inhibited ADP-ribosylation of Gαi in CHO-K1 and A549 cells which subsequently, caused decreased cAMP signaling in the living cell-based interference in the Gαi-mediated signal transduction (iGIST) assay. When analyzing the mode of inhibition mediated by α-defensin-1 and − 5, it was shown that both α-defensins but not β-defensin-1 inhibited the enzyme activity of PTS1 in vitro. In addition, α-defensin-1 and − 5 showed inhibition of binding and uptake of PT into cells, while the inhibitory effect was stronger for α-defensin-5. It was shown that α-defensin-1, but not α-defensin-5 is taken up into different cell lines and interacts with PTS1 inside cells. This was supported by in silico modeling, demonstrating the α-defensin-1 but not α-defensin-5 contains specific interaction interfaces with PTS1, while this was further confirmed using dot blot experiments. Therefore, the inhibition of PT by α-defensin-1 was mostly mediated via inhibition of enzyme activity of PTS1, and α-defensin-5 mainly inhibited cellular uptake of PT into target cells. In summary, α-defensin-1 and − 5 are also promising candidates for the treatment of pertussis.

### α_1_AT and derived peptides as inhibitors for PT, C2 toxin, DT, and anthrax fusion toxin

Recently, by employing bioassay-guided fractionation and mass spectrometry analysis during screening of a human hemofiltrate library, α_1_AT was identified as inhibitor for PT from *B. pertussis* (Lietz et al. 2024). Also others have identified α_1_AT as endogenous inhibitor through a screening approach of a human derived BAL library, however as inhibitor of SARS-CoV-2 virus entry via inhibition of TMPRSS2 (Wettstein et al. 2021). The endogenous serin protease inhibitor α_1_AT (52 kDa) is as a member of the serpin-family.

To analyze the inhibitory mode of action of α_1_AT on PT, biochemistry-, cell culture-, and molecular modeling-based in vitro experiments, as well the in vivo infant mouse model were used. The inhibitory capabilities of α_1_AT were based on the direct interaction of α_1_AT most likely with the binding subunit of PT which inhibited PT binding to target cells, including CHO-K1 cells and the more physiological relevant lung adenoma cell line A549 cells. In vivo the application of α_1_AT in the infant mouse model of pertussis, significantly reduced pertussis-induced leukocytosis, the hallmark of pertussis, in *B. pertussis* infected mice treated with α_1_AT in direct comparison to control mice which were infected with *B. pertussis* but not treated with α_1_AT. Interestingly, murine mRNA levels for α_1_AT were reduced in *B. pertussis* infected mice. To our knowledge, so far, it is not known whether severe pertussis is associated with reduced levels of α_1_AT and whether individuals with low α_1_AT levels are more susceptible for developing severe pertussis outcome.

Moreover, α_1_AT also has inhibitory activity towards related bacterial AB-type protein toxins and was found to additionally inhibit C2 toxin from *C. botulinum*, DT from *C. diphtheriae*, and an *B. anthracis* fusion toxin, revealing α_1_AT as multi-toxin inhibitor (Lietz et al. 2024). The characterization of the mode of inhibition of C2 toxin by α_1_AT revealed that α_1_AT inhibited C2 toxin in a dual mode of action via inhibition of C2 toxin binding to cells and the enzyme activity of C2I in vitro. Besides, α_1_AT interacts most likely with the binding subunits of C2 toxin and anthrax toxin, C2IIa and PA_63_ respectively.

Interestingly, α_1_AT-containing drugs are already approved medications and clinically applied as augmentation therapy for the treatment of α_1_AT-deficiency. As such, α_1_AT-containing drugs such as Prolastin^®^ should be investigated for the treatment of pertussis and other toxin-mediated diseases including diphtheria and anthrax in reproposing approaches.

Most recently, to further explore α_1_AT as inhibitor of PT, the amino acid region that bears anti-PT activity was investigated (Lietz et al. 2025). Therefore, α_1_AT derived peptides were generated and tested for their ability to inhibit PT. Additionally, the α_1_AT derived peptide, termed VIRIP which was previously identified from a human hemofiltrate library as inhibitor of HIV-1 (Münch et al. 2007) showed no inhibition of PT. However, the three α_1_AT derived peptides termed α_1_AT-HF, 42, and 64 were identified to inhibit the ADP-ribosylation of Gαi in PT-treated CHO-K1 cells. These three α_1_AT derived peptides share a common sequence, while α_1_AT-HF which is endogenously present in hemofiltrate has the shortest sequence of α_1_AT with anti-PT activity. Additionally, the endogenous α_1_AT-HF peptides showed no adverse effects on cell viability and in vivo in zebrafish embryos.

### Angie peptides as inhibitors for TcdA and TcdB

Lately, the in silico predicted Angie peptides derived from the human, endogenous protein angiogenin were identified as inhibitors for the glucosylating toxins, TcdA and TcdB from *C. difficile* (Lietz et al. 2025). The Angie peptides (Angie 1, 3, 5, 6, 7, and reference) have a size of 17 amino acids and vary only by one or two amino acids at positions 2 and 5 or 12. Here, the strongest inhibitory capacity of all tested Angie peptides was provided by Angie 5, followed by Angie 1 and 3, consistently in three different cell lines, HeLa, Vero, and the more physiologically relevant human colon carcinoma cell line CaCo-2. The other Angie peptides, Angie 6, 7, and the reference Angie showed no inhibition of TcdA, TcdB, and the combination of both toxins. Moreover, Angie 5 showed a concentration- and time-dependent inhibition of TcdB-mediated glucosylation of Rac-1. When analyzing the mode of inhibition, the Angie peptides showed no inhibition of toxin binding to target cells, no aggregate formation, and no inhibition of auto-protease activity, and no inhibition of enzyme activity. Therefore, the mode of inhibition must be related to other steps of toxin trafficking within target cells, e.g. the translocation of the toxin from the endosomes into the cytosol. In silico modeling of the TcdB-Angie 5 complex showed that the hydrophobic N-terminus of Angie 5 is attributed a core function in mediating the stabilization of the TcdB-Angie 5 complex located in the hydrophobic pocket formed at the interface between the GTD, CPD, and DRBD domains. Consequently, the ability to inhibit TcdA and TcdB is strongly connected to the amino acid composition of the Angie peptides.

Previously, the human protein angiogenin and the derived peptide termed Angie 1 were found to possess antimicrobial properties against *M. tuberculosis* (Noschka et al. 2021). Therefore, the Angie peptides were tested for their ability to inhibit the ESKAPE pathogens, including *Pseudomonas (P.) aeruginosa*, *Acinetobacter (A.) baumannii*,* Escherichia (E.) coli*, *Enterococcus (E.) faecium*, *Staphylococcus (S.) aureus*, and *Klebsiella (K.) pneumoniae*. Interestingly, the antimicrobial Angie peptides, Angie 1, 3 and 5, were also identified to be inhibitors of different other bacteria, including *C. difficile*, *P. aeruginosa*, and *A. baumannii*. The hydrophobic, cationic properties of Angie 1, 3, and 5 most likely enable the interaction and disruption of the anionic bacterial cell membranes. When analyzing of *C. difficile* bacteria incubated with Angie 5 with transmission electron microscopy, disrupted cell membranes were observed compared to bacteria incubated with the solvent control (water). Consequently, the Angie peptides are another example of peptides with antimicrobial properties that are also efficient inhibitors of bacterial AB-type protein toxins, such as TcdA and TcdB.

### Non-human derived peptide inhibitors for bacterial AB-type protein toxins

Despite our approach to identify inhibitors for bacterial AB-type toxins from human sources, others used phage display screenings or in silico algorithms to identify or specifically design peptide-based inhibitors for a concrete binding pocket of AB-type toxins, e.g., the catalytic domain.

Abdeen and colleagues used a screening of 7-mer peptides to identify candidates that bind the active site of TcdA to inhibit its enzymatic activity and therefore the glucosylation of target proteins (Abdeen et al. 2010). In detail, the authors used a phage display approach where they screened a commercially available PhD7 (NEB) library for peptides that specifically bind the recombinant catalytic fragment of TcdA (rTcdA^540^). The assay was designed to specifically select for peptides binding to the substrate binding pocket of rTcdA and thus identified peptides that are in direct competition with the substrate, RhoA. This resulted in the discovery of 36 unique peptides from in total 200 screened peptide sequences, while lead candidates were further selected based on the affinity of peptides towards TcdA. In comparison to RhoA, the lead candidates showed tight binding to TcdA and both lead peptides inhibited TcdA and TcdB in a nanomolar range.

One of the lead sequences identified by Abdeen and colleagues was later used by others as a reference sequence (RP) during an in silico approach to identify peptide-based inhibitors for TcdA (Xiao et al. 2022). Here, a Monte Carlo-based peptide binding design (PepBD) algorithm was developed and used to identify two 10-mer TcdA neutralizing peptides A and B (NPA and NPB). These two in silico predicted peptides, were subsequently experimentally validated using a cytopathic cell rounding assay, where the RP, NPA, and NPB protected human primary jejunum cells from TcdA-mediated cytotoxic effects.

Moving forward, the same research group used the PepBD algorithm to find shorter peptides that bind to the catalytic GTD domain of TcdA based on previously identified 10-mer peptide NPA and to optimize these peptides to bind TcdA GTD with higher binding affinity. This led to the in silico identification of the 8-mer SA1 peptide which protected human colonic epithelium from TcdA-mediated effects.

Moreover, Larabee and colleagues identified peptides derived of the amino acid region of TcdB that affects epitope exposure and cytotoxicity which protect cells from TcdB-mediated cytotoxic effects (Larabee et al. 2017). The lead candidate PepB2 was characterized for its mode of TcdB inhibition using different in vitro and cell-based assays. Through a various set of methods, they could show that PepB2 does not inhibit the enzyme activity of TcdB and rather interacts with the CROP region of TcdB which leads to the formation of polymeric complexes and the destabilization of TcdB.

Similar approaches as for TcdA and TcdB have been performed for anthrax toxin, cholera toxin, and shiga toxin (Table 2).

**Table 2:** Examples for non-human derived peptides with anti-toxin activity

Toxin	Bacterium	Investigated inhibitors	Screening methods	Mechanism of inhibition	References
TcdA, TcdB	*C. difficile*	EGWHAHT and HQSPWHH (lead candidates, other sequences were identified)	Phage display approach, PhD7 (NEB) library	Binding and inhibition of catalytic domains of TcdA and TcdB	(Abdeen et al. 2010)
TcdA	*C. difficile*	DYWFQRHGHR (NPA), GMFWQHRRHD (NPB), EGWHAHTGGG (RP) (lead candidates, other sequences were identified)	In silico approach, Monte Carlo-based peptide binding design (PepBD) algorithm	Binding and inhibition of catalytic domains of TcdA	(Xiao et al. 2022)
TcdA	*C. difficile*	DYWFQRHG (NPA-derived), EFWWRRHN (SA1) (lead candidates, other sequences were identified)	In silico approach, Monte Carlo-based peptide binding design (PepBD) algorithm	Binding and inhibition of catalytic domains of TcdA	(Sarma et al. 2023)
TcdB	*C. difficile*	SA1	In silico approach, Monte Carlo-based peptide binding design (PepBD) algorithm	Low binding affinity for catalytic domain of TcdB	(Sarma et al. 2023)
TcdB	*C. difficile*	RPDQRTAP (LSB1), QRPQQRTF (LSB5), QRPAPQTR (LSB6), and optimized candidates derived from LSB5 (SB2, SB5, SB6)	Solid phase library screening, *de novo* peptide discovery	Binding and inhibition of catalytic domain of TcdB	(Catella et al. 2025)
TcdB	*C. difficile*	NVFKGNTISDKISFNFSDK (PepB2) (lead candidate, other sequences were identified)	Intrinsic toxin-derived peptides of TcdB	Binding to CROP domain of TcdB, polymeric complex formation, and TcdB destabilization	(Larabee et al. 2017)
TcdA	*C. difficile*	PepB2	Intrinsic toxin-derived peptides of TcdB	No inhibition	(Larabee et al. 2017)
LT	*B. anthracis*	PepB2	Intrinsic toxin-derived peptides of TcdB	No inhibition	(Larabee et al. 2017)
LF/ LF_N_DTA /EF + [PA_63_]_7_	*B. anthracis*	HTSTYWWLDGAP (P1), HQLPQYWWLSPG (P2) (generation of polyvalent inhibitors)	Phage display approach, PhD12 (NEB) library	Binding of peptide to PA_63_ heptamer blocking interaction with EF and LF, anthrax protection in vitro and in vivo	(Mourez et al. 2001), subsequent studies with core peptide motive or P1 (Glick et al. 2002; Hicks et al. 2004)
LF_(N)_ + [PA_63_]_7_	*B. anthracis*	TYWWLD (lead candidate, other sequences were identified)	Phage display approach, PhD7 (NEB) library	Binding of peptides to surface of PA_63_ heptamer blocking toxin assembly	(Gujraty et al. 2005)
LF/EF + [PA_63_]_7_	*B. anthracis*	TLPYWWLTPSNP (p2), NVMTYWWLDPPL (p3) (synthesis of peptides as linear (p2 and p3) and tetra-branched form (MAP2 and MAP3), other sequences were identified) NAMTYWWLDPPL (MAP3 V/A) YWWLTPPP	Phage display approach, PhD12 (NEB) library	Inhibition of PA63-LF binding Inhibition of EF-induced cAMP increase and protection in vivo after PA + LF treatment of rats No inhibition of EF-induced cAMP increase	(Pini et al. 2006)
CT	*V. cholerae*	GGRHRRR (GGR-tet), GNRHRRR (NRR-tet) (other sequences were identified)	Affinity-based screening of tetravalent random-peptide libraries	Binding of peptides to receptor-binding region of CT (B-pentamer)	(Watanabe-Takahashi et al. 2024)
Stx2	*E. coli*	PPPRRRR (PPP-tet) (lead candidate, other sequences were identified)	Screening of tetravalent peptide library	Aberrant cellular transport and degradation of Stx2	(Nishikawa et al. 2006)
Stx1, Stx2	*E. coli*	MMARRRR (MMA-tet) (lead candidate, other sequences were identified)	Screening of tetravalent peptide library	Rescue of Stx1 induced inhibition of protein synthesis	(Tsutsuki et al. 2013)
Stx2a, Stx2d	*E. coli*	LMARRRR (LMA-tet), QMARRRR (QMA-tet), IMARRRR (IMA-tet), MMVRRRR (MMV-tet), MMMRRRR (MMM-tet) (other sequences were tested)	Screening of tetravalent peptide library	Binding of Stx2 B-subunits	(Mitsui et al. 2016)
Stx1a, Stx2a	*E. coli*	MMβARRRR (MMβA-tet) (lead candidate, other sequences were identified) MMβA-mono	Screening of tetravalent peptides	Targeting of Stx1 B-subunit through multivalent interaction by tetravalent peptide, peptide monomer inhibited cytotoxicity by binding to A-subunit	(Watanabe-Takahashi et al. 2021)
Stx2	*E. coli*	TFNMWLPTFNQW (TF-1), WAPWYSFTSYHL (WA-8) (lead candidates, other sequences were identified)	Phage display approach, 12-mer library (NEB)	Inhibition of Stx2 binding to target cells	(Li et al. 2016)

## Conclusion

Many highly infectious diseases are caused by bacteria that produce symptom-triggering AB-type protein toxins. Recently, for those infectious diseases, including pertussis and diphtheria, increasing case numbers with several breakouts and deaths have been reported, also in Western countries (European Centre for Disease Prevention and Control, 2023; European Centre for Disease Prevention and Control., 2024, 2025).

Bacterial AB-type protein toxins have evolved a very sophisticated uptake mechanism, by which they are able to enter mammalian target cells. After receptor-binding enabled by the B-domain, the B-domain facilitates the translocation of the enzymatically active A-domain from cellular compartments into the cytosol. Here, the A-domain executes its toxin-specific enzymatic reaction which leads to further cellular reactions that cause the characteristic symptoms of the disease. Therefore, the bacterial toxins are directly responsible for the development of the symptoms after bacterial infections. Consequently, the inhibition of bacterial toxins would prevent the development of symptoms. For some bacterial infections there are preventive measures, such as the vaccinations for e.g., pertussis and diphtheria and symptomatic treatment options including antibiotics available. However, for the treatment of toxin-mediated diseases, there are no specific and targeted curative therapeutics available.

Therefore, novel toxin-targeted strategies to combat toxin-mediated diseases are urgently required for patient treatment in the clinics. Currently, often antibiotics are prescribed for infectious diseases, including pertussis, diphtheria, anthrax, and for CDIs. However, antibiotics are only effective at an early stage of e.g., pertussis to have beneficial effects on disease progression of the patient (Mattoo and Cherry 2005). Moreover, treatment with antibiotics can also directly be the root cause of the disease e.g., in case of CDIs, which can also occur due to disrupted gut microbiome after antibiotic treatment (Spigaglia 2024).

For the identification of novel inhibitors for bacterial AB-type protein toxins, huge potential lies within the human proteome/peptidome. The human body is constantly exposed to a broad set of pathogens and therefore might has developed strategies as part of the innate immune system to fight against bacterial AB-type protein toxins. As a result, human derived protein/peptide libraries might resemble a rich source of protein/peptides-based toxin inhibitors. The systematic screening of these protein/peptide libraries might lead to the identification of novel inhibitors of bacterial AB-type protein toxins. Moreover, the screening of proteins/peptides with antimicrobial activity, including ABPs and AVPs, might lead to the identification of novel inhibitors of bacterial AB-type protein toxins. The unraveling of protein/peptide characteristics that are required for the inhibition of bacterial toxins might also contribute to the in silico selection, prediction, and optimization of potential protein/peptide-based inhibitors.

So far, several endogenous proteins/peptides were already identified either from screening approaches or testing of proteins/peptides that possess antimicrobial activity. Currently, the largest group of endogenous toxin inhibitors is resembled by the human defensins, especially by the α-defensin. The α-defensin have a broad antimicrobial activity and are also endogenous inhibitors against multiple bacterial toxins, predominantly, against iota toxin (Fischer et al. 2018), the clostridial toxins CDT, TcdA, and TcdB (Barthold et al. 2022; Fischer et al. 2020; Giesemann et al. 2008; Korbmacher et al. 2020), diphtheria toxin (Kim et al. 2006), anthrax toxin (Giesemann et al. 2008; Kim et al. 2005; Wei et al. 2009), and PT (Kling et al. 2021, 2023). Furthermore, HSA was identified as inhibitor for TcdA and TcdB (di Masi et al. 2018; Tonon et al. 2020). Moreover, the human serin protease inhibitor α_1_AT was identified from screening of a human hemofiltrate library as inhibitor for PT, C2 toxin, DT, and an anthrax fusion toxin (Lietz et al. 2024; Lietz et al. 2024). In addition, also peptides derived from α_1_AT, 42, 64, and the endogenous peptide α_1_AT-HF were identified as inhibitors of PT (Lietz et al. 2025). Finally, the angiogenin derived Angie peptides which also possess antimicrobial activity against *M. tuberculosis* but also against *C. difficile* (Lietz et al. 2025; Noschka et al. 2021) were identified as inhibitors of TcdA and TcdB (Lietz et al. 2025).

Since several endogenous proteins/peptides were identified to possess anti-toxin activity for a set of different clinically relevant toxin-mediated diseases, those inhibitors should be explored in further studies including in vivo studies. For example, α_1_AT-containing drugs are already approved for the treatment of the α_1_AT deficiency, they could be more easily explored in an off-label application for the treatment and management of pertussis. Moreover, ABPs such as the Angie peptides inhibit the *C. difficile* bacterium and its produced toxins, TcdA and TcdB at the same time. This resembles a dual mode of action which could be advantageous for treatment of patients since it would be superior to antibiotics which only target the bacteria but not its released toxins. Therefore, especially proteins/peptides with antibacterial and toxin activity might be a promising approach for the treatment of infectious diseases in the future.

The successful identification of human endogenous protein/peptides as natural inhibitors of bacterial toxins underlines and highlights the role of the innate immune system to fight not only bacteria but also its produced virulence factors. Finally, endogenous proteins/peptides have huge potential to be toxin inhibitors, while by far the complete potential has not been reached and many potential inhibitors of bacterial toxins still need to be identified and explored for their mechanism of action.

## Abbreviations

α_1_AT: α_1_-antitrypsin
Gαi: α-subunit of inhibitory G-proteins subunit of inhibitory G-proteins
*A.**baumannii*: *Acinetobacter baumannii*
ART: ADP-ribosyltransferase
AC: Adenylate cyclase
*A.** hydrophila*: *Aeromonas* *hydrophila*
ACE2: Angiotensin-converting enzyme 2
ABPs: Antibacterial peptides
AMPs: Antimicrobial peptides
AVPs: Antiviral peptides
*B.**anthracis*: *Bacillus* *anthracis*
*B.** pertussis*: *Bordetella* *pertussis*
BAL: Bronchoalveolar lavage
CDADs: *C.* *difficile*-associated diseases
CDI: *C.* *difficile* infections
CGTs: Clostridial glucosylating toxins
*C.** difficile*: *Clostridioides* *difficile*
*C.** botulinum*: *Clostridium botulinum*
*C.** perfringens*: *Clostridium* *perfringens*
*C.** sordellii*: *Clostridium* *sordellii*
CHO-K1 cells: Chinese hamster ovary cells strain K1
CDCs: Cholesterol-dependent cytolysins
CaCo-2: Colorectal adenocarcinoma cells 2
*C*. *diphtheriae*: *Corynebacterium* *diphtheriae*
cAMP: Cyclic AMP
CPD: Cysteine protease domain
def: Defensin
DT: Diphtheria toxin
DTA: Diphtheria toxin enzyme subunit
DTB: Diphtheria toxin binding subunit
EF: Edema factor
EF-2: Elongation factor 2
ER: Endoplasmic reticulum
*E.** faecium*: *Enterococcus faecium*
ERAD: ER-associated degradation
*E.**coli*: *Escherichia coli*
ESKAPE pathogens: (*Pseudomonas *(*P.*)* aeruginosa, Acinetobacter *(*A.*)* baumannii, Escherichia *(*E.*)* coli, Enterococcus *(*E.*)* faecium, Staphylococcus *(*S.*)* aureus, and Klebsiella *(*K.*)* pneumoniae*)
ECDC: European centre for disease prevention and control
ETA: Exotoxin A
GPCRs: G-protein-coupled receptors
GTD: Glucosyltransferase domain
IC_50_: Half-maximal inhibitory concentration
Hsp90: Heat shock protein 90
HB-EGF: Heparin-binding epidermal growth factor-like growth factor precursor
HSA: Human serum albumin
InsP6: Inositol hexakisphosphate
iGIST: Interference in Gαi-mediated signal transduction
*K.** pneumoniae*: *Klebsiella pneumoniae*
LF: Lethal factor
LPS: Lipopolysaccharides
MALDI-TOF: Matrix-assisted laser desorption ionization-time of flight
*M.** tuberculosis*: *Mycobacterium* *tuberculosis*
*N.** gonorrhoeae*: *Neisseria* *gonorrhoeae*
PVL: Panton-Valentine leukocidin
PT: Pertussis toxin
PA: Protective antigen
*P.** aeruginosa*: *Pseudomonas* *aeruginosa*
*S.** aureus*: *Staphylococcus aureus*
TcdA: Toxin A
TcdB: Toxin B
TEER: Trans-epithelial electrical resistance
V-ATPases: Vesicular adenosine triphosphatases
*V.** cholerae*: *Vibrio* *cholerae*
